# Biallelic mutations in RNA-binding protein ADAD2 cause spermiogenic failure and non-obstructive azoospermia in humans

**DOI:** 10.1093/hropen/hoad022

**Published:** 2023-05-18

**Authors:** Baolu Shi, Wasim Shah, Li Liu, Chenjia Gong, Jianteng Zhou, Tanveer Abbas, Hui Ma, Huan Zhang, Menglei Yang, Yuanwei Zhang, Nadeem Ullah, Zubair Mahammad, Mazhar Khan, Ghulam Murtaza, Asim Ali, Ranjha Khan, Jiahao Sha, Yan Yuan, Qinghua Shi

**Affiliations:** Division of Reproduction and Genetics, First Affiliated Hospital of USTC, School of Basic Medical Sciences, Division of Life Sciences and Medicine, Biomedical Sciences and Health Laboratory of Anhui Province, University of Science and Technology of China, Hefei, China; Division of Reproduction and Genetics, First Affiliated Hospital of USTC, School of Basic Medical Sciences, Division of Life Sciences and Medicine, Biomedical Sciences and Health Laboratory of Anhui Province, University of Science and Technology of China, Hefei, China; State Key Laboratory of Reproductive Medicine, Nanjing Medical University, Nanjing, Jiangsu, China; Division of Reproduction and Genetics, First Affiliated Hospital of USTC, School of Basic Medical Sciences, Division of Life Sciences and Medicine, Biomedical Sciences and Health Laboratory of Anhui Province, University of Science and Technology of China, Hefei, China; Division of Reproduction and Genetics, First Affiliated Hospital of USTC, School of Basic Medical Sciences, Division of Life Sciences and Medicine, Biomedical Sciences and Health Laboratory of Anhui Province, University of Science and Technology of China, Hefei, China; Division of Reproduction and Genetics, First Affiliated Hospital of USTC, School of Basic Medical Sciences, Division of Life Sciences and Medicine, Biomedical Sciences and Health Laboratory of Anhui Province, University of Science and Technology of China, Hefei, China; Division of Reproduction and Genetics, First Affiliated Hospital of USTC, School of Basic Medical Sciences, Division of Life Sciences and Medicine, Biomedical Sciences and Health Laboratory of Anhui Province, University of Science and Technology of China, Hefei, China; Division of Reproduction and Genetics, First Affiliated Hospital of USTC, School of Basic Medical Sciences, Division of Life Sciences and Medicine, Biomedical Sciences and Health Laboratory of Anhui Province, University of Science and Technology of China, Hefei, China; Division of Reproduction and Genetics, First Affiliated Hospital of USTC, School of Basic Medical Sciences, Division of Life Sciences and Medicine, Biomedical Sciences and Health Laboratory of Anhui Province, University of Science and Technology of China, Hefei, China; Division of Reproduction and Genetics, First Affiliated Hospital of USTC, School of Basic Medical Sciences, Division of Life Sciences and Medicine, Biomedical Sciences and Health Laboratory of Anhui Province, University of Science and Technology of China, Hefei, China; Division of Reproduction and Genetics, First Affiliated Hospital of USTC, School of Basic Medical Sciences, Division of Life Sciences and Medicine, Biomedical Sciences and Health Laboratory of Anhui Province, University of Science and Technology of China, Hefei, China; Division of Reproduction and Genetics, First Affiliated Hospital of USTC, School of Basic Medical Sciences, Division of Life Sciences and Medicine, Biomedical Sciences and Health Laboratory of Anhui Province, University of Science and Technology of China, Hefei, China; Division of Reproduction and Genetics, First Affiliated Hospital of USTC, School of Basic Medical Sciences, Division of Life Sciences and Medicine, Biomedical Sciences and Health Laboratory of Anhui Province, University of Science and Technology of China, Hefei, China; Division of Reproduction and Genetics, First Affiliated Hospital of USTC, School of Basic Medical Sciences, Division of Life Sciences and Medicine, Biomedical Sciences and Health Laboratory of Anhui Province, University of Science and Technology of China, Hefei, China; Division of Reproduction and Genetics, First Affiliated Hospital of USTC, School of Basic Medical Sciences, Division of Life Sciences and Medicine, Biomedical Sciences and Health Laboratory of Anhui Province, University of Science and Technology of China, Hefei, China; Division of Reproduction and Genetics, First Affiliated Hospital of USTC, School of Basic Medical Sciences, Division of Life Sciences and Medicine, Biomedical Sciences and Health Laboratory of Anhui Province, University of Science and Technology of China, Hefei, China; State Key Laboratory of Reproductive Medicine, Nanjing Medical University, Nanjing, Jiangsu, China; State Key Laboratory of Reproductive Medicine, Nanjing Medical University, Nanjing, Jiangsu, China; Division of Reproduction and Genetics, First Affiliated Hospital of USTC, School of Basic Medical Sciences, Division of Life Sciences and Medicine, Biomedical Sciences and Health Laboratory of Anhui Province, University of Science and Technology of China, Hefei, China; Institute of Health and Medicine, Hefei Comprehensive National Science Center, Hefei, China

**Keywords:** non-obstructive azoospermia, ADAD2, spermiogenesis, round spermatid injection, genetic counselling

## Abstract

**STUDY QUESTION:**

What are some pathogenic mutations for non-obstructive azoospermia (NOA) and their effects on spermatogenesis?

**SUMMARY ANSWER:**

Biallelic missense and frameshift mutations in *ADAD2* disrupt the differentiation of round spermatids to spermatozoa causing azoospermia in humans and mice.

**WHAT IS KNOWN ALREADY:**

NOA is the most severe cause of male infertility characterized by an absence of sperm in the ejaculate due to impairment of spermatogenesis. In mice, the lack of the RNA-binding protein ADAD2 leads to a complete absence of sperm in epididymides due to failure of spemiogenesis, but the spermatogenic effects of *ADAD2* mutations in human NOA-associated infertility require functional verification.

**STUDY DESIGN, SIZE, DURATION:**

Six infertile male patients from three unrelated families were diagnosed with NOA at local hospitals in Pakistan based on infertility history, sex hormone levels, two semen analyses and scrotal ultrasound. Testicular biopsies were performed in two of the six patients. *Adad2* mutant mice (*Adad2^Mut/Mut^*) carrying mutations similar to those found in NOA patients were generated using the CRISPR/Cas9 genome editing tool. Reproductive phenotypes of *Adad2^Mut/Mut^* mice were verified at 2 months of age. Round spermatids from the littermates of wild-type (WT) and *Adad2^Mut/Mut^* mice were randomly selected and injected into stimulated WT oocytes. This round spermatid injection (ROSI) procedure was conducted with three biological replicates and >400 ROSI-derived zygotes were evaluated. The fertility of the ROSI-derived progeny was evaluated for three months in four *Adad2^WT/Mut^* male mice and six *Adad2^WT/Mut^* female mice. A total of 120 *Adad2^Mut/Mut^*, *Adad2^WT/Mut^*, and WT mice were used in this study. The entire study was conducted over 3 years.

**PARTICIPANTS/MATERIALS, SETTING, METHODS:**

Whole-exome sequencing was performed to detect potentially pathogenic mutations in the six NOA-affected patients. The pathogenicity of the identified *ADAD2* mutations was assessed and validated in human testicular tissues and in mouse models recapitulating the mutations in the NOA patients using quantitative PCR, western blotting, hematoxylin-eosin staining, Periodic acid-Schiff staining, and immunofluorescence. Round spermatids of WT and *Adad2^Mut/Mut^* mice were collected by fluorescence-activated cell sorting and injected into stimulated WT oocytes. The development of ROSI-derived offspring was evaluated in the embryonic and postnatal stages.

**MAIN RESULTS AND THE ROLE OF CHANCE:**

Three recessive mutations were identified in *ADAD2* (MT1: c.G829T, p.G277C; MT2: c.G1192A, p.D398N; MT3: c.917_918del, p.Q306Rfs*43) in patients from three unrelated Pakistani families. MT1 and MT2 dramatically reduced the testicular expression of ADAD2, likely causing spermiogenesis failure in the NOA patients. Immunofluorescence analysis of the *Adad2^Mut/Mut^* male mice with the corresponding MT3 mutation showed instability and premature degradation of the ADAD2 protein, resulting in the spermiogenesis deficiency phenotype. Through ROSI, the *Adad2^Mut/Mut^* mice could produce pups with comparable embryonic development (46.7% in *Adad2^Mut/Mut^* versus 50% in WT) and birth rates (21.45 ± 10.43% in *Adad2^Mut/Mut^* versus 27.5 ± 3.536% in WT, *P* = 0.5044) to WT mice. The *Adad2^WT/Mut^* progeny from ROSI (17 pups in total via three ROSI replicates) did not show overt developmental defects and had normal fertility.

**LARGE SCALE DATA:**

N/A.

**LIMITATIONS, REASONS FOR CAUTION:**

This is a preliminary report suggesting that ROSI can be an effective treatment for infertile *Adad2^Mut/Mut^* mice. Further assisted reproductive attempts need to be carefully examined in humans during clinical trials.

**WIDER IMPLICATIONS OF THE FINDINGS:**

Our work provides functional evidence that mutations in the *ADAD2* gene are deleterious and cause consistent spermiogenic defects in both humans and mice. In addition, preliminary results show that ROSI can help *Adad2^Mut/Mut^* to produce biological progeny. These findings provide valuable clues for genetic counselling on the *ADAD2* mutants-associated infertility in human males.

**STUDY FUNDING/COMPETING INTEREST(S):**

This work was supported by the National Natural Science Foundation of China (32000587, U21A20204, and 32061143006), and the National Key Research and Developmental Program of China (2019YFA0802600 and 2021YFC2700202). This work was also supported by Institute of Health and Medicine, Hefei Comprehensive National Science Center, Hefei, China. The authors declare no competing interests.

WHAT DOES THIS MEAN FOR PATIENTS? Non-obstructive azoospermia (NOA) is characterized by impaired spermatogenesis and is the most severe form of male infertility. Approximately 25% of NOA cases are attributed to genetic anomalies, but only mutations in a small number of genes have been validated as pathogenic in NOA patients.

Our study focused on six infertile patients with NOA who were carriers of three *ADAD2* mutations from different and unrelated families. Functional evidence has shown that these mutations cause premature degradation of the ADAD2 protein and are associated with failure of round spermatids to differentiate into spermatozoa during spermatogenesis.

Despite the lack of spermatozoa in the testes, *Adad2^Mut/Mut^* mice with mutation efficiencies similar to that of human patients were able to produce healthy and fertile offspring after round spermatid injection. Therefore, our study provides a preliminary insight for the genetic counselling of couples with *ADAD2*-mutation-associated male infertility.

## Introduction

Spermatogenesis is a highly coordinated process that involves spermatogonial proliferation and differentiation, meiotic division of spermatocytes and post-meiotic differentiation from round spermatids to spermatozoa (also termed spermiogenesis) (de Kretser *et al.*, 1998). Impairment of any step in spermatogenesis can cause non-obstructive azoospermia (NOA), which accounts for 20% of infertility in men (Jiang *et al.*, 2022). Although genetic anomalies have been identified in about 25% of NOA cases (Krausz and Riera-Escamilla, 2018), mutations in only 14 genes have been verified to cause NOA (Sudhakar *et al.*, 2021; Jiang *et al.*, 2022).

Round spermatid injection (ROSI), an ART, involves the injection of round spermatids (derived from testicular biopsies) into the recipient’s oocytes (Tesarik *et al.*, 1995). In NOA patients with round spermatids as the most mature germ cells in the testes, ROSI is considered the last resort for the production of biological offspring (Tesarik *et al.*, 1995; Antinori *et al.*, 1997; Barak *et al.*, 1998; Gianaroli *et al.*, 1999; Tanaka *et al.*, 2015, 2018).

ADAD2 is a double-stranded RNA-binding protein that is expressed exclusively in the testis (Wang *et al.*, 2015; Snyder *et al.*, 2020). Male mice lacking ADAD2 (herein referred to as *Adad2^ko^*) are infertile with a complete absence of sperm in the epididymides due to defective spermiogenesis (Snyder *et al.*, 2020; Chukrallah *et al.*, 2022). Previous studies have suggested that a homozygous stop-gain mutation in *ADAD2* (c.1186C>T, p.Gln396Ter) and compound heterozygous mutations (Hg19: chr16:84012049–84224913del and c.82dupC, p.Gln28ProfsTer136) in *ADAD2* might be associated with incomplete spermatogonial arrest (Krausz *et al.*, 2020). Patients with severe asthenoteratozoospermia carrying a homozygous frameshift mutation (c.17_18del, p.Gln6Argfs*3) or a homozygous missense mutation (c.1381C>T, p.Arg461Trp) in *ADAD2* showed morphological deformities in both the sperm head and flagellum assembly (Dai *et al.*, 2023; Tian *et al.*, 2023), suggesting that ADAD2 may affect human spermatogenesis. However, the reproductive phenotype of these *ADAD2* mutations identified in humans differ from those in *Adad2^ko^* mice, and the pathological effects of these *ADAD2* mutations have not been verified in mouse models. Therefore, the functional role of *ADAD2* in human spermatogenesis and testicular spermatogenic defects due to *ADAD2* mutations in infertile men require further exploration.

In this study, we identified three *ADAD2* mutations (MT1, MT2, and MT3) in six NOA patients from Pakistan. Patients harboring biallelic MT1 or MT2 mutation had significantly reduced levels of testicular ADAD2 protein and defects in spermiogenesis, which is consistent with the observation in *Adad2^ko^* mice. Male *Adad2^Mut/Mut^* mice corresponding to MT3 in men had similar infertile phenotypes. Importantly, although the round spermatids of *Adad2^Mut/Mut^* displayed aberrant chromatin organization, ROSI helped them produce fertile offspring. Thus, our study provides direct *in vivo* and *ex vivo* evidence that biallelic mutations in *ADAD2* cause NOA in humans, and ROSI is a feasible treatment for the *Adad2^Mut/Mut^* mice which have mutational efficiency similar to that of *ADAD2*-mutated NOA patients.

## Materials and methods

### Clinical samples

In this study, we recruited six infertile men from three unrelated families who were diagnosed with idiopathic NOA. Four patients, including two brothers in Family 1 and two brothers in Family 3, were born to consanguineous parents. All the patients had normal height and secondary sexual characteristics but failed to produce offspring even after trying to conceive during >6 years of marriage (Table 1). All the patients had normal karyotypes (46, XY) and no Y-chromosome microdeletions (Table 1). Serum levels of reproductive hormones including FSH, luteinizing hormone (LH), testosterone, and estradiol were in the normal range, as measured by local laboratories (Table 1). Testicular shape and outline were normal, while a reduction in the bilateral testicular volume was observed in five patients (Table 1). Semen analysis was performed twice with the 12-week interval between the analyses for each patient, in accordance with the WHO guidelines (World Health Organization, 2021); all six men ejaculated the normal volume of semen, which contained no sperm (Table 1).

**Table 1.** Clinical characteristics of six NOA patients with mutations in *ADAD2*.

Subject	Family 1 (c.G829T)		Family 2 (c.G829T/c.G1192A)		Family 3 (c.917_918del)		Reference values
	IV-1	IV-6	III-3	III-4	IV-1	IV-2	
**Basic information**							
Reproductive status	Infertility	Infertility	Infertility	Infertility	Infertility	Infertility	–
Age (y)^a^	26	34	29	27	35	33	–
Body mass index (BMI)	23.8	25.6	30	31.2	24.8	24.2	–
Age (y) of marriage	20	19	19	19	25	25	–
**Genetic testing**							
Karyotype	46, XY	46, XY	46, XY	46, XY	46, XY	46, XY	
Y-chromosomal microdeletions	Negative	Negative	Negative	Negative	Negative	Negative	
**Semen parameters** ^b^ ^,^ ^d^							
Semen volume (ml)—first test	4.5	3.0	4.0	2.6	2.9	2.2	>1.3
Semen volume (ml)—second test	2.1	2.2	2.0	2.2	2.1	1.6	
Sperm concentration (10^6^/ml)—first and second tests	0	0	0	0	0	0	>15
**Physical examination** ^c^							
External genitalia	Normal	Normal	Normal	Normal	Normal	Normal	–
Secondary traits	Normal	Normal	Normal	Normal	Normal	Normal	–
**Ultrasonography** ^e^							
Left testis size (cm^3^)	6.0	7.0	6.2	–	8.5	9.0	>12.5
Right testis size (cm^3^)	6.3	7.6	6.5	–	8.0	11.5	
**Hormone concentrations** ^e^							
FSH, U/l	6.2	9.4	–	6.5	3.6	–	1.7–12.0
LH, U/l	6.4	5.2	–	5.0	1.9	–	1.7–8.6
Testosterone, ng/ml	8.8	4.6	–	3.5	5.5	–	2.3–10.3
Estradiol, pmol/l	174.9	66.2	–	146.8	168.0	–	19.7–242.0

a Ages at the manuscript submission.

b Semen analysis was performed twice for each infertile individual following the WHO guidelines (World Health Organization, 2021).

c Physical examination was performed by the local andrologist.

d Reference values of semen parameters and testicular size were published in the WHO laboratory manual for the examination and processing of human semen (World Health Organization, 2021).

e Reference values were suggested by the local hospital or clinical laboratory.

The controls in this study were men who had been diagnosed with obstructive azoospermia (OA) with no obvious defects in spermatogenesis. Written informed consent was received from all participants prior to the onset of the study. The study was approved by the institutional ethics committee of the University of Science and Technology of China (USTC) with the approval number 2019-KY-168.

### Whole-exome sequencing and mutation selection

Whole-exome sequencing (WES) and mutation selection were performed as reported previously (Zhang *et al.*, 2020; Fan *et al.*, 2021). Briefly, total genomic DNA was isolated from the peripheral blood samples. Whole-exome capture and sequencing were performed using the AlExome Enrichment Kit V1 (iGeneTech, Beijing, China) and Hiseq2000 platform (Illumina, San Diego, CA, USA), following standard procedures. The reads were aligned to the human genome reference assembly (hg19) using Burrows–Wheeler Aligner with default parameters. PCR duplicates were removed by the Picard software (http://picard.sourceforge.net/). DNA mutation sequences were analyzed using Genome Analysis Toolkit HaplotypeCaller (http://www.broadinstitute.org/gatk/). Mutations that met the following filtration criteria were subjected to further analyses to consider the following: (i) mutations that could alter protein sequence; (ii) mutations with minor allele frequency <0.01 in the 1000 Genomes, ESP6500, ExAC, and Genome Aggregation Database, and were absent as homozygous or compound heterozygous in our in-house WES data sets from 578 fertile men; (iii) nonsense, frameshift and splice mutations, and missense deleterious mutations as predicted by at least half of the used software: Sorting Intolerant From Tolerant (SIFT), PolyPhen2 HDIV, MutationTaster, MutationAssessor, fathmm_MKL, GERP++, and SiPhy; (iv) mutations in genes that are expressed in the testis; (v) mutations following recessive inheritance patterns including autosome recessive, compound heterozygous and sex-linked recessive patterns; and (v) mutations in the spermatogenesis-related genes predicted by the SpermatogenesisOnline database (Zhang *et al.*, 2013) or verified in an animal model. For the consanguineous families (Families 1 and 3), mutations associated with recessive inheritance patterns and located in Regions of Homozygosity (RoHs) were prioritized, while compound heterozygous mutations were preferred from the non-consanguineous family (Family 2). The homozygosity mapping analysis was performed using Bcftools (Narasimhan *et al.*, 2016).

### Sanger sequencing

*ADAD2* variants were detected by Sanger sequencing of all available family members and mice. GoldenStar^®^ T6 Super PCR Mix (1.1×) (Tsingke Biotechnology TSE101, Beijing, China) was used for PCR and the reactions were performed as follows: 2 min at 98°C, 38 cycles of 20 s at 98°C, 30 s at 55°C, and 30 s at 72°C. The obtained PCR products were electrophoresed in 2% agarose gels to check for the correct band size before Sanger sequencing. The primer sequences used are listed in Supplementary Table S1.

### Histological analysis

Human and mouse testicular tissues were fixed overnight in Bouin’s solution, rinsed with 50% ethanol for 5 min, and then dehydrated in an ethanol gradient (70%, 80%, 90%, and 100%, 20 min for each). The tissues were then transferred into xylene twice for 15 min each time and were immersed three times in paraffin (45 min for each immersion). Finally, the tissues were embedded in paraffin and cut into 5-μm sections using a Leica RM2235 Manual Rotary Microtome (Leica Biosystems, Germany). For hematoxylin–eosin (H&E) staining, the tissue sections were first deparaffinized in xylene, rehydrated with gradient ethanol, and stained sequentially with hematoxylin (Solarbio Life Sciences G1140, Shanghai, China) and eosin (Solarbio Life Sciences 15086-94-9). The sections were then rinsed with increasing ethanol concentrations and incubated in xylene before sealing with neutral resin (Sinopharm Chemical Reagent 96949-21-2, Shanghai, China). For Periodic acid-Schiff (PAS) staining, sections were deparaffinized, rehydrated, and then stained with the PAS reagent (Yuanye Bio-Technology R20526, Shanghai, China) before counterstaining with hematoxylin. After dehydration and clearing, the tissue sections were sealed with neutral resin. The images were captured on a Nikon ECLIPSE 80i microscope (Nikon Instruments, Tokyo, Japan) with a DS-Ri1 camera and processed by NIS-Elements Basic Research software (Nikon Instruments). The image acquisition parameters are listed in Supplementary Table S2.

### Generation of *Adad2^Mut/Mut^* mice

*Adad2^Mut/Mut^* mice carrying the mutation analogous to that identified in the NOA patients (*ADAD2* c.917_918del) were generated using CRISPR/Cas9 genome editing tools. A single guide RNA (sgRNA) targeting the mouse *Adad2* genomic sequence close to the corresponding mutation site was designed using the webserver: http://crispor.tefor.net/. The sgRNA and Cas9 protein were transferred into C57BL/6 zygotes by electroporation. The founder mice mutations were confirmed by Sanger genomic DNA sequencing. The founder heterozygous *Adad2* mutant mice were bred with 8-week-old C57BL/6 WT mice (GemPharmatech, Nanjing, China) to produce heterozygous F1 mice. Homozygotes were obtained by the inter-crossing of heterozygous mice from the third backcross. The primers used for the generation and genotyping of *Adad2-*mutant mice are listed in Supplementary Table S1. All animals were housed in a specific-pathogen-free animal facility with a 12h:12h light:dark cycle. All animal studies were conducted following the guidelines of the Institutional Animal Care Committee of USTC (approval number USTCACUC1301021) and the Institutional Animal Care and Use Committees of Nanjing Medical University (IACUC-2009002).

### Epidydimal sperm count

The unilateral epididymides of WT or *Adad2^Mut/Mut^* mice were removed and cut into small pieces that were transferred to an Eppendorf tube containing 1 ml of 1× PBS (68.5 mM NaCl, 1.3 mM KCl, 5.0 mM Na_2_HPO_4,_ and 0.9 mM KH_2_PO_4_). After incubation for 30 min at 37°C, sperm were released into the PBS. Then, 10 µl of the PBS sperm suspension (diluted 10 times in 1× PBS) were placed on a hemocytometer and the sperm samples were counted under the microscope. All the mouse samples had three replicates which were averaged to obtain the final value.

### Staging of seminiferous tubules

The staging of mouse seminiferous tubule sections with immunofluorescence (Russell *et al.*, 1990; Shi *et al.*, 2019; Gao *et al.*, 2020), H&E (Ahmed and de Rooij, 2009), and PAS (Ahmed and de Rooij, 2009; Meistrich and Hess, 2013) staining was determined based on previous reports.

### RNA extraction and qPCR

Total RNA was extracted from the testes as we described previously (Zhang *et al.*, 2020; Gong *et al.*, 2022). Briefly, total RNA was extracted with TRIzol (Accurate Biology AG21101, Hunan, China) and the cDNAs were synthesized with the PrimeScript RT reagent kit with gDNA Eraser (TaKaRa RR047A, Kusatsu, Japan). The concentration and purity of cDNA were measured using a NanoDrop 1000 Spectrophotometer (Thermo Fisher Scientific). The qPCR was performed with FastStart Universal SYBR Green Master (ROX) (Roche 04913850001, Basel, Switzerland) on a Step One Real-Time PCR System (Applied Biosystems, Thermo Fisher Scientific). The qPCR reactions were performed under the following conditions: 10 min at 95°C, 40 cycles of 10 s at 95°C and 30 s at 60°C. The qPCR data were analyzed by the 2^−ΔΔCt^ method (Schmittgen and Livak, 2008); *Actb* (NM_007393.5) was the internal control. The primer sequences used are listed in Supplementary Table S1.

### Immunofluorescent staining of paraffin-embedded sections

Mouse or human testes were fixed in 4% paraformaldehyde overnight, then embedded in paraffin and cut into 5-μm sections. For immunofluorescence-staining, slides were dewaxed, rehydrated, permeabilized, and then transferred to citrate-based antigen retrieval solution (0.3% trisodium citrate dihydrate and 0.04% citric acid monohydrate in ddH_2_O). Afterward, the slides were heated at 96°C for 20 min and blocked with BDT solution (3% bovine serum albumin and 10% normal donkey serum in 1× TBST composed of 50 mM Tris (pH 7.4), 150 mM NaCl, and 0.1% Tween-20). The tissue slides were then incubated with primary antibodies, followed by secondary antibodies, and mounted with VECTASHIELD Antifade Mounting Medium (Vector Laboratories H-1000, San Francisco, CA, USA) containing 5 μg/ml Hoechst 33342 (Invitrogen H1399, Thermo Fisher Scientific). Images were captured using an Olympus BX53 Microscope (Tokyo, Japan) with cellSens imaging software. The Image-Pro Plus software (MEDIA CYBERNETICS, USA) was used for HP1α-positive foci counting. The image acquisition parameters are listed in Supplementary Table S2. The antibodies used are listed in Supplementary Table S3.

### Western blotting

Human testicular proteins were extracted from tissue lysates prepared using TRIzol (Accurate Biology AG21101, Hunan, China) as we reported previously (Yin *et al.*, 2019). The 1 ml of TRIzol was added to human testicular tissue, and the sample homogenization was performed on ice. The lysate was then added to 200 µl of chloroform and mixed thoroughly before centrifugation at 12 000×*g* for 15 min at 4°C. After centrifugation, the upper aqueous phase was removed and 300 μl of 100% ethanol was added to the interphase and organic phase, before thorough mixing and centrifugation at 2000× *g* for 5 min at 4°C. The supernatant was precipitated with 1.5 ml of isopropanol by centrifugation at 12 000× *g* for 10 min at 4°C. The new supernatant was discarded, and 2 ml of cleaner solution (0.3 M guanidine hydrochloride in 95% ethanol) was added to the precipitate. After incubation for 20 min, the mixture was centrifuged at 7500× *g* for 5 min at 4°C. The precipitate was then thoroughly mixed with 2 ml of 100% ethanol and again centrifuged at 7500× *g* for 5 min at 4°C. This final precipitate contained the human testicular proteins, which were dissolved in 200 μl of 1% SDS and heated at 50°C for 30 min and denatured at 100°C for 10 min for subsequent western blotting.

Protein extracts from mice testes were prepared in lysis buffer containing 50 mM Tris (pH 7.5), 150 mM NaCl, 0.5% Triton X-100, 2.5 mM EDTA, and 1× phenylmethylsulfonyl fluoride protease inhibitor (Thermo Scientific 36978, Waltham, MA, USA). The extracts were sonicated and then centrifuged. After heat denaturation, the proteins were separated by SDS-PAGE and then transferred onto 0.45-μm nitrocellulose membranes (GE Healthcare Life Sciences 10600002, Pittsburgh, PA, USA). The membranes were blocked in 5% nonfat milk and incubated with primary antibodies followed by incubation with secondary antibodies. Finally, the blots were developed for chemiluminescence (GE Healthcare Life Sciences ImageQuant LAS 4000). The antibodies used are listed in Supplementary Table S3.

The blots were quantified using Image J software (National Institutes of Health).

### Oocyte collection

CD1 female mice (GemPharmatech, Nanjing, China), 3–4 weeks old, were induced to superovulate by i.p. injection of 7.5 IU Pregnant Mare Serum Gonadotropin (EasyCheck M2620, Nanjing, China) and then 7.5 IU HCG (EasyCheck M2520) after 46–48 h. Oocytes were collected from oviducts 12–14 h after HCG injection and the cumulus was digested with 0.2% bovine testicular hyaluronidase (Sigma-Aldrich H4272, Merck, Germany) in M2 medium (Sigma-Aldrich MR-015-D) for 2–3 min. The released oocytes were transferred to fresh M2 medium at 37°C under 5% CO_2_.

### Flow cytometry analysis for haploid cells isolation

Testes from C57BL/6J WT (littermates of *Adad2^Mut/Mut^* mice) and *Adad2^Mut/Mut^* male mice (8–10 weeks old) were isolated. After removal of the tunica, the seminiferous tubules were cut into small pieces, placed in Dulbecco’s Modified Eagle Medium (DMEM) (Gibco 11965092, Thermo Fisher Scientific), and digested with 1 mg/ml collagenase (Sigma-Aldrich C4-BIOC) for 15 min, followed by 0.25% trypsin–EDTA (Gibco 25200072) digestion for 10 min. The germ cell suspension was incubated with Hoechst 33342 (Invitrogen, 62249) at a concentration of 5 μg/ml for 15 min at 37°C and sorted on the BD FACSAria Fusion SOP system (BD Biosciences, USA) equipped with BD FACSDiva software (BD Biosciences). Two-way sorting was performed using a 100 μm nozzle size. The flow sorting rate was 3000 events/s. Hoechst dye was excited using a 355-nm laser, and the dye’s wide emission spectrum was detected in Hoechst Blue (450/50 nm band-pass filter). Forward Scatter (FSC-A) and Side Scatter (SSC-A) were detected using a 488-nm laser. Haploid cells (1N) were collected based on the peaks of the cell population at 1N DNA content (Bastos *et al.*, 2005; Barroca *et al.*, 2009; Gaysinskaya *et al.*, 2014). The sorted population of haploid cells was collected in 4 ml of DMEM in 15 ml tubes and centrifuged at 200× *g* for 10 min. Then haploid round spermatid cells (1N) were selected from the obtained cell pellet under a microscope for subsequent oocyte injection.

### Round spermatid injection and embryo transfer

The procedure of oocyte stimulation has been described elsewhere (Li *et al.*, 2012). Before microinjection, the MII oocytes were pre-stimulated in Ca^2+^-free CZB medium (EasyCheck M0000, Nanjing, China) containing 10 mM SrCl_2_ (Sigma-Aldrich 439665) for 10 min, followed by a 5 min exposure to M2 medium containing 5 µg/ml cytochalasin B (Sigma-Aldrich C6762). An individual round spermatid cell was injected into a pre-stimulated oocyte with a Piezo-driven pipette. After injection, embryos were activated in Ca^2+^-free CZB medium containing 10 mM SrCl_2_ at 37°C under 5% CO_2_ for 3–5 h. Finally, the injected embryos were transferred into fresh KSOM medium (Sigma-Aldrich MR-101-D) for *in vitro* culture. Different stages of embryos were collected at 40 (two-cell stage), 54 (four-cell stage), 84 (morula), and 96 (blastocyst) hours post-HCG injection for counting and imaging. Two-cell stage embryos were collected and transferred into the oviduct of CD1 pseudopregnant females. Full-term pups were delivered naturally.

### Statistical analysis

GraphPad Prism software (GraphPad Software, San Diego, CA, USA) was used to perform the statistical analyses. Data are presented as mean ± SD in Figs 3C, E and 4B, D, F–G, and Supplementary Figs 3B–D and 4A, B. Student’s *t*-test was used for all statistical analyses. The data with *P-*values <0.05 were considered significant.

## Results

### Identification of *ADAD2* mutations in three unrelated Pakistani families

**Figure 1.**
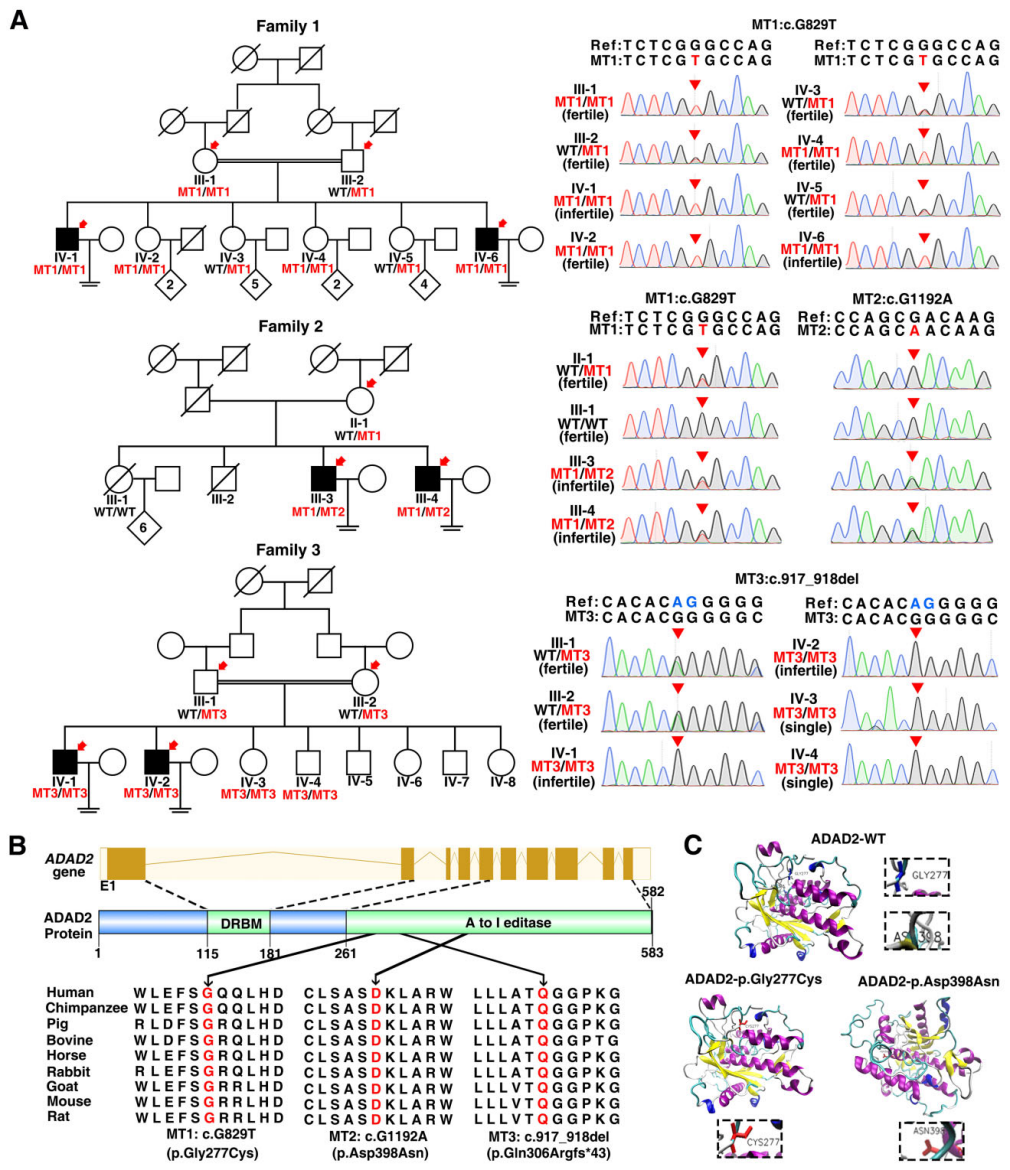
**Identification of *ADAD2* mutations in non-obstructive azoospermia (NOA) affected men from three unrelated Pakistani families.** (**A**) Identification of *ADAD2* mutations from three unrelated Pakistani families. Whole-exome sequencing was performed on members indicated by red arrows. Double horizontal lines represent the consanguineous marriages. Squares and circles denote male and female members, separately. Solid symbols indicate the infertile males, and open symbols denote unaffected members. Slashes represent deceased family members. The genotypes of available members which were validated by Sanger sequencing are labeled below the symbols. Sanger sequencing chromatograms of the *ADAD2* mutations in all infertile males, their available siblings and parent(s) are shown on the right. The red arrowheads indicate the mutation sites. The mutant nucleotides (MT1 and MT2) and the deleted nucleotides (MT3) are labeled in red and blue, respectively. (**B**) Schematic representing the locations of *ADAD*2 mutations at genome and protein levels, and the conservation analysis of affected amino acids in different organisms. The yellow solid rectangles represent exons, and the yellow lines represent introns. The schematic gene and protein structure are drawn based on the Ensembl database (GRCh38, transcript ID: ENST00000315906.10) and UniProtKB (access ID: Q8NCV1). (**C**) Predicted spatial structures of the ADAD2 protein in native conformation and conformational changes caused by the mutations. MT1 (p.Gly277Cys) resulted in the replacement of Glycine by a buried Cysteine. MT2 (p.Asp398Asn) replaced a charged Aspartic acid residue with an uncharged residue Asparagine and disrupted the salt bridge. Amino acid substitutions caused by MT1 and MT2 led to structural change of ADAD2 adenosine deaminase domain. The prediction software was SWISSMODEL4.0 (http://missense3d.bc.ic.ac.uk/).

WES was performed to identify potential candidate genes associated with NOA in the three unrelated Pakistani families, including all six infertile patients, their siblings and parent(s). The obtained mutations were subsequently screened by a series of criteria (Supplementary Fig. S1 and Table S4). Consequently, recessive mutations in *ADAD2* were identified in the three Pakistani families (Fig. 1A). In Family 1, Patients IV-1 and IV-6 harbored a homozygous missense mutation in *ADAD2* (MT1: c.G829T, p.Gly277Cys). In Family 2, Patients III-3 and III-4 harbored *ADAD2* compound heterozygous missense mutations (MT1: c.G829T, p.Gly277Cys and MT2: c.G1192A, p.Asp398Asn). In Family 3, a homozygous 2-bp-deletion in *ADAD2* (MT3: c.917_918del, p.Gln306Argfs*43) was identified in Patients IV-1 and IV-2. The allele frequencies of all these three mutations are below 0.01% in human populations (Table 2). The MT1 and MT2 mutations were predicted to be deleterious by SIFT, PolyPhen2, MutationTaster, and fathmm-MKL tools (Table 2). The identified *ADAD2* mutations were further verified by Sanger sequencing and found to be co-segregated with male infertility in the respective families (Fig. 1A).

**Table 2.** ADAD2 variants identified in three unrelated Pakistani families.

Subjects		Genomic position on chr16 (bp)	cDNA mutation	Protein alteration	Mutation type	Affected allele	SIFT	Polyphen2	Mutation taster	fathmm-MKL	1KGP AF	gnomAD AF
Family 1	MT1	84 228 997	c.G829T	p.Gly277Cys	Missense	Homozygous	D	D	D	D	0	0.00002128
Family 2	MT1	84 228 997	c.G829T	p.Gly277Cys	Missense	Compound heterozygous	D	D	D	D	0	0.00002128
	MT2	84 229 560	c.G1192A	p.Asp398Asn	Missense		D	D	D	D	0	0
Family 3	MT3	84 229 167	c.917_918del	p.Gln306Argfs*43	Frameshift	Homozygous	NA	NA	NA	NA	0	0.00009528

NCBI accession number of *ADAD2* is NM_001145400.2. 1KGP, 1000 Genomes Project; chr, chromosome; gnomAD, the Genome Aggregation Database; AF, allele frequency; D, deleterious; NA, not available.

ADAD2 (also known as TENRL, GenBank: NM_001145400.2) is an RNA-binding protein that is specifically expressed in the testes of humans and mice. The three *ADAD2* mutations were located in its adenosine deaminase domain, which is highly conserved across species (Fig. 1B). Furthermore, both MT1 and MT2 were predicted to alter the conformation of the adenosine deaminase domain (Fig. 1C). The MT3 mutation introduced a premature stop codon and was predicted to cause protein truncation or non-sense mediated mRNA decay. ADAD2 is reported to be essential for mouse spermatogenesis as *Adad2^ko^* male mice are infertile due to spermiogenesis failure (Snyder *et al.*, 2020). Thus, we speculated that these identified mutations in *ADAD2* are likely the cause of male infertility in the three families.

### *ADAD2* missense mutations dramatically reduced ADAD2 protein levels in families 1 and 2

**Figure 2.**
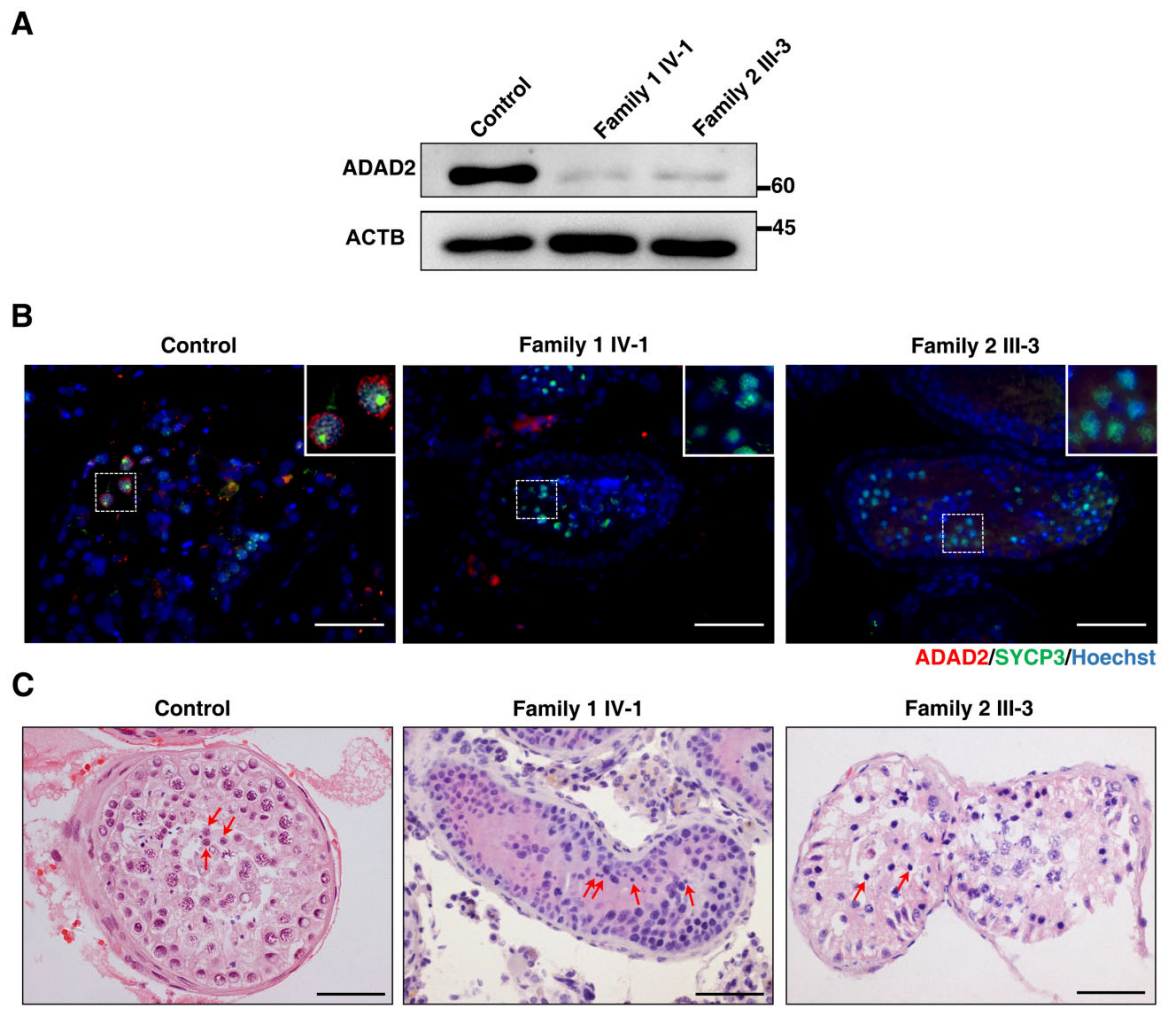
**Effects of *ADAD2* mutations on ADAD2 protein expression and spermatogenesis in patients.** (**A**) Western blot (WB) analysis of ADAD2 expression in testicular tissues of an obstructive azoospermia (OA) male control and men harboring *ADAD2* variants. ACTB was used as an internal control. (**B**) Immunofluorescence staining of ADAD2 (red) and synaptonemal complex protein 3 (SYCP3) (green) in human testicular section of an OA control and men harboring *ADAD2* variants. Magnified views of cytoplasmic localization pattern of ADAD2 are shown in the upper right corner of the overlay images. Scale bar, 50 μm. (**C**) Hematoxylin and eosin (H&E) of testicular cross-sections from an OA control and infertile patients carrying *ADAD2* variants. The red arrows point to round spermatids which were differentiated according to previous reports (Clermont, 1963; Nihi *et al.*, 2017). Scale bars, 50 μm.

To investigate changes in the expression level of ADAD2 protein due to *ADAD2* mutation, we performed western blotting, and a protein band of the expected size was detected in testicular samples from a control OA man who had normal spermatogenesis. Patient IV-1 from Family 1 (homozygous for MT1) and Patient III-3 from Family 2 (with compound heterozygous MT1 and MT2) had significantly lower levels of ADAD2 protein, seen as only trace amounts of the protein in these patients (Fig. 2A). Furthermore, immunofluorescence staining was barely able to detect ADAD2 in IV-1 and III-3 testicular samples while the protein was present in the spermatocyte cytoplasm from the OA control (Fig. 2B). Thus, the *ADAD2* MT1 and MT2 mutations largely reduced the level of ADAD2 protein in the testes of the affected patients.

### Defects in spermiogenesis due to *ADAD2* mutations

To further assess the impact of the *ADAD2* mutations on spermatogenesis, testicular biopsies of the IV-I (Family 1) and III-3 (Family 2) patients were assessed by H&E staining. Histological analysis revealed the presence of spermatogonia, spermatocytes, and round spermatids in the seminiferous tubules of IV-I and III-3, which were comparable with that of the control OA patient (Fig. 2C). However, few spermatozoa could be seen in the patients’ samples (Fig. 2C), suggesting defects in spermiogenesis in both patients. Considering that the spermiogenic defects observed in the IV-I and III-3 patients were highly similar to those in *Adad2^KO^* mice (Snyder *et al.*, 2020), we considered that the MT1 and MT2 missense mutations were deleterious and were likely the genetic cause of NOA in Families 1 and 2.

### *Adad2^Mut/Mut^* male mice are infertile and mimic the spermiogenic defects of *ADAD2* mutation-carrying men

Considering that the ADAD2 protein level was observed to be markedly reduced in the testes of the patients carrying MT1 and MT2 (Fig. 2A), and pathological histology showed defective spermiogenesis (Fig. 2C) consistent with the phenotype of *Adad2^KO^* mice (Snyder *et al.*, 2020), we hypothesized that MT1 and MT2 were associated with male infertility. As the two patients carrying MT3 (c.917_918del, p.Gln306Argfs*43) in Family 3 did not agree to the testicular biopsy, the pathogenicity of the MT3 mutation could not be verified in patient samples, and we therefore generated a mouse model (*Adad2^Mut/Mut^*) carrying a frameshift mutation (c.851insA, p.Pro287Serfs*41) that was corresponded to the human MT3 mutation (Supplementary Fig. S2A and C). The predicted truncated ADAD2 protein in the patients carrying the MT3 mutation and *Adad2^Mut/Mut^* mouse contains both the whole double-stranded RNA-binding (DBRM) domain and the shortened adenosine deaminase domain containing similar numbers of residues (Supplementary Fig. S2B).

**Figure 3.**
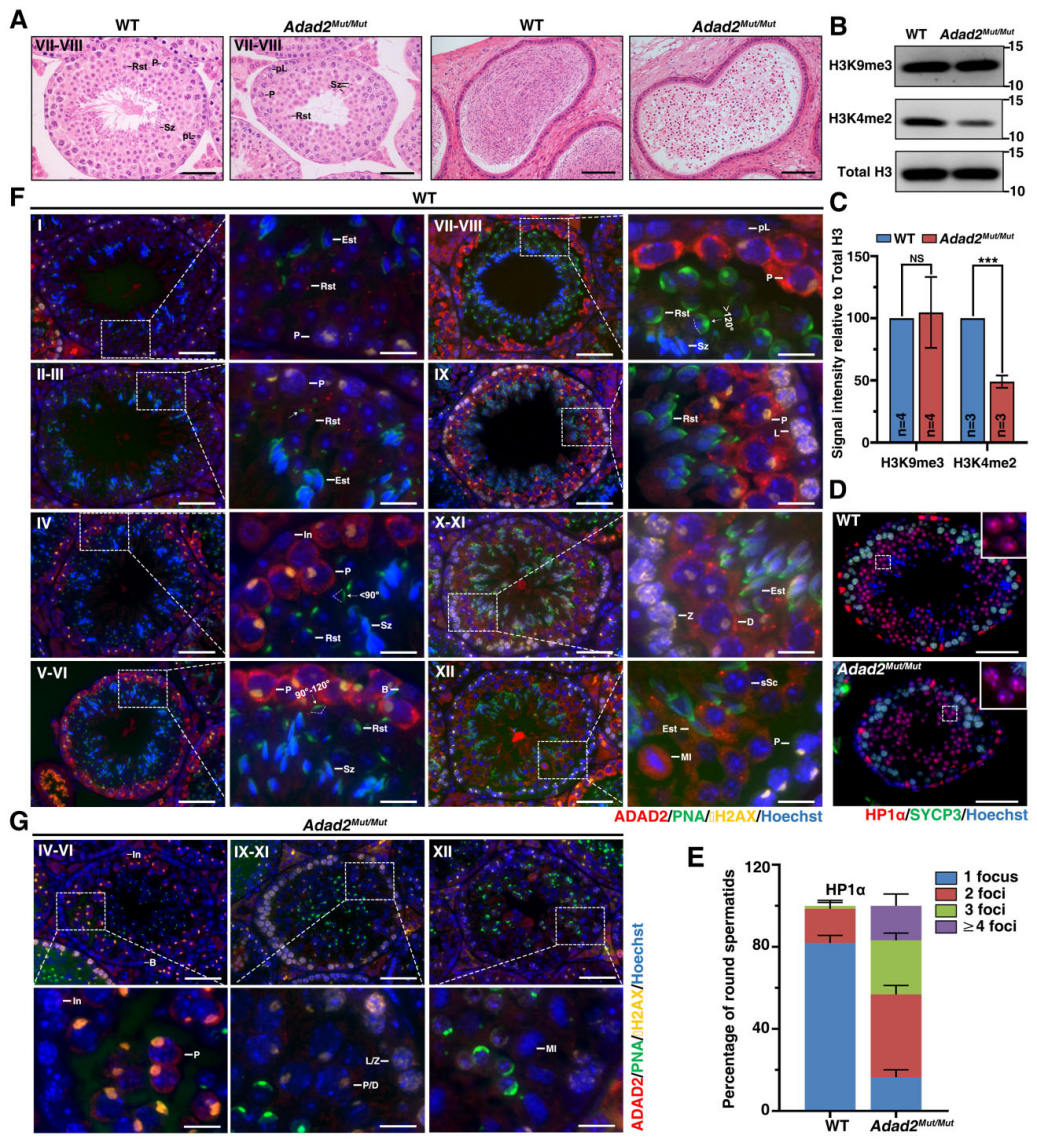
**Spermatogenic analyses of *Adad2^Mut/Mut^* mice**. (**A**) Representative images of Stage VII–VIII seminiferous tubules and epididymis of WT and *Adad2^Mut/Mut^* mice. Testicular and epididymal cross-sections were stained by hematoxylin and eosin (H&E). Staging of mouse seminiferous tubule referred to Ahmed and de Rooij (2009). pL, preleptotene; P, pachytene; Rst, round spermatid; Sz, spermatozoa. Scale bars, 50 μm (testis) and 100 μm (epididymis). (**B**) Western blot (WB) analysis of H3K9me3 and H3K4me2 expression in testes from 8-week-old WT and *Adad2^Mut/Mut^* mice. Total H3 was used as a loading control. WB was performed in triplicates. (**C**) Signal intensity of H3K9me3 and H3K4me2. Data are presented as mean ± SD. ***P < 0.001, NS, no significance, unpaired t test. n indicates the number of repetitions for each group. (**D**) Immunofluorescence staining of HP1α (red) and synaptonemal complex protein 3 (SYCP3) (green). Magnified views of the HP1α foci in round spermatids are shown in the upper right corner of the overlay images. Scale bars, 50 μm. (**E**) Quantification of the HP1α foci in round spermatids at Stage IV tubules. Three pairs of 8-week-old WT and *Adad2^Mut/Mut^* littermates were analyzed; error bars indicate SD. Immunofluorescence staining of the testicular sections of 8-week-old WT (**F**) and *Adad2^Mut/Mut^* littermates (**G**) using ADAD2, acrosome marker peanut agglutinin (PNA), and DNA double stranded breaks marker γH2AX. DNA was counterstained with Hoechst. Staging of testicular sections was based on previous reports (Russell *et al.*, 1990; Nakata *et al.*, 2015; Shi *et al.*, 2019; Gao *et al.*, 2020). White arrows indicate the angle of acrosomes. Magnified views of the ADAD2 signal in spermatogenic cells are shown on the right. In, intermediate spermatogonia; B, type B spermatogonia; pL, preleptotene; L, leptotene; Z, zygotene; P, pachytene; D, diplotene; MI, metaphase I; sSC, secondary spermatocyte; Rst, round spermatid; Est, elongating spermatid (in Stage IX–XI tubules) or elongated spermatid (in Stage XII and I–III tubules); Sz, spermatozoa. Scale bars, 50 and 12.5 μm (enlarged box).

Similar to the *ADAD2*-mutation-carrying patients, the *Adad2^Mut/Mut^* male mice were infertile (Supplementary Fig. S3D) with significant reductions in testicular size (Supplementary Fig. S3A and B) and an absence of spermatozoa in the epididymides (Fig. 3A; Supplementary Fig. S3C). H&E staining revealed that the seminiferous tubules of both WT and *Adad2^Mut/Mut^* mice contained preletotene spermatocytes and multiple layers of round spermatids at Stage VII–VIII (Fig. 3A). However, unlike the WT mice which had a layer of spermatozoa lining in the lumen, only a few spermatozoa were observed in the *Adad2^Mut/Mut^* mice (Fig. 3A), suggesting that spermiogenesis was compromised in the *Adad2^Mut/Mut^* mice. We then used PAS staining to investigate the spermiogenesis defects in more detail. Mouse seminiferous tubules can be divided into 12 stages (Russell *et al.*, 1990). No obvious morphological defects in the Stage I–VIII tubules were seen in the *Adad2^Mut/Mut^* mice (Supplementary Fig. S3E). From Stage IX, the round spermatids in WT mice began to elongate. However, this process was largely delayed in the *Adad2^Mut/Mut^* mice, with most of the spermatids having insufficiently flattened nuclei at Stage XII, and even spermatids without elongation were also visible (Supplementary Fig. S3E). The number of elongating spermatids gradually decreased from Stage XII, and consequently, few spermatozoa were observed in the lumens of Stage VII–VIII tubules in the *Adad2^Mut/Mut^* mice (Supplementary Fig. S3E). These findings indicate that the *Adad2^Mut/Mut^* mice have defective spermiogenesis due to *ADAD2* mutations and suggest that similar mutations (MT3) in *ADAD2* could cause human male infertility.

The absence of the ADAD2 protein in *Adad2^ko^* mice has been shown to result in aberrant chromatin organization in meiotic spermatocytes and post-meiotic spermatids, characterized by increased heterochromatin marking at H3K9me3 and heterochromatin protein 1α (HP1α), and reduced euchromatin marking at H3K4me2 (Chukrallah *et al.*, 2022). To investigate whether *Adad2* mutations affect the chromatin status in *Adad2^Mut/Mut^* mice, we performed the same chromatin marking analysis in the WT and *Adad2^Mut/Mut^* mice. We found that compared with the WT mice, the level of H3K9me3 marking was not affected but the level of H3K4me2 marking was significantly reduced in the testes of the *Adad2^Mut/Mut^* mice (Fig. 3B and C). In addition, consistent with the reported *Adad2^KO^* mice, immunostaining of HP1α showed multiple HP1α foci in the nuclei of round spermatids from the *Adad2^Mut/Mut^* mice that were not seen in the WT mice (Fig. 3D and E). This suggests that defective heterochromatin distribution may be responsible for the aberrant differentiation of round spermatids in the *Adad2^Mut/Mut^* mice.

### Reduction of ADAD2 protein in the testes of *Adad2^Mut/Mut^* male mice

To explore the effects of *Adad2^Mut/Mut^* mutations in mice, qPCR analysis was performed, and the relative expression of *Adad2* mRNA between the testes of 3-week-old WT and *Adad2^Mut/Mut^* littermates showed that *Adad2* expression was reduced by 40% in *Adad2^Mut/Mut^* (Supplementary Fig. S4A). Moreover, immunofluorescence staining was used to examine whether the mutation led to the translation of the truncated ADAD2 protein. In WT mice, the ADAD2 protein was detected in Stage IV pachytene spermatocytes, where it was well-dispersed in the cytoplasm. The immunofluorescence signal of the protein subsequently aggregated to form perinuclear granules in the cytoplasm of mid- and late-pachytene spermatocytes from Stage V to IX tubules (Fig. 3F). Bright perinuclear granules were observed in diplotene spermatocytes at Stages X and XI as well as secondary spermatocytes at Stage XII (Fig. 3F) and foci-like ADAD2 was still present in the cytoplasm of round spermatids in early steps in Stage I–VI tubules (Fig. 3F) but was completely absent during the following stages (Fig. 3F). The staging of the seminiferous tubules in the *Adad2^Mut/Mut^* mice was difficult to determine using the acrosome marker peanut agglutinin (PNA), as the transition from round to elongated spermatids was largely delayed (Supplementary Fig. S3E). Therefore, we roughly classified the staging in the *Adad2^Mut/Mut^* mice using the marker for DNA double-strand breaks, γH_2_AX, to distinguish the different phases of meiotic spermatocytes (Shi *et al.*, 2019) and Hoechst 33342 to examine the type of spermatogonia (Russell *et al.*, 1990). Cytoplasmic ADAD2 signals were detected in pachytene spermatocytes but were much weaker than those in the WT in Stage IV–VI tubules which contained intermediate and type-B spermatogonia (Fig. 3G); the exposure time of the ADAD2 signal was the same as that used for the WT. Stage IX–XI tubules were characterized by nuclei filled with γH_2_AX (leptotene/zygotene cells) along the basal membrane, and ADAD2 signals were completely absent from pachytene or diplotene spermatocytes (Fig. 3G). The ADAD2 signals also disappeared from Metaphase I and secondary spermatocytes in Stage XII tubules (Fig. 3G). No ADAD2 staining was found in round spermatids from any of the tubules (Fig. 3G). Given that the level of *Adad2* mRNA was close to the background level at diplotene (Supplementary Fig. S4B) (Chen *et al.*, 2018), the absence of the ADAD2 protein in *Adad2^Mut/Mut^* diplotene spermatocytes suggests that a truncated ADAD2 protein (which would be less stable) was produced but degraded rapidly.

### ROSI helped produce healthy offspring from *Adad2^Mut/Mut^* male mice

**Figure 4.**
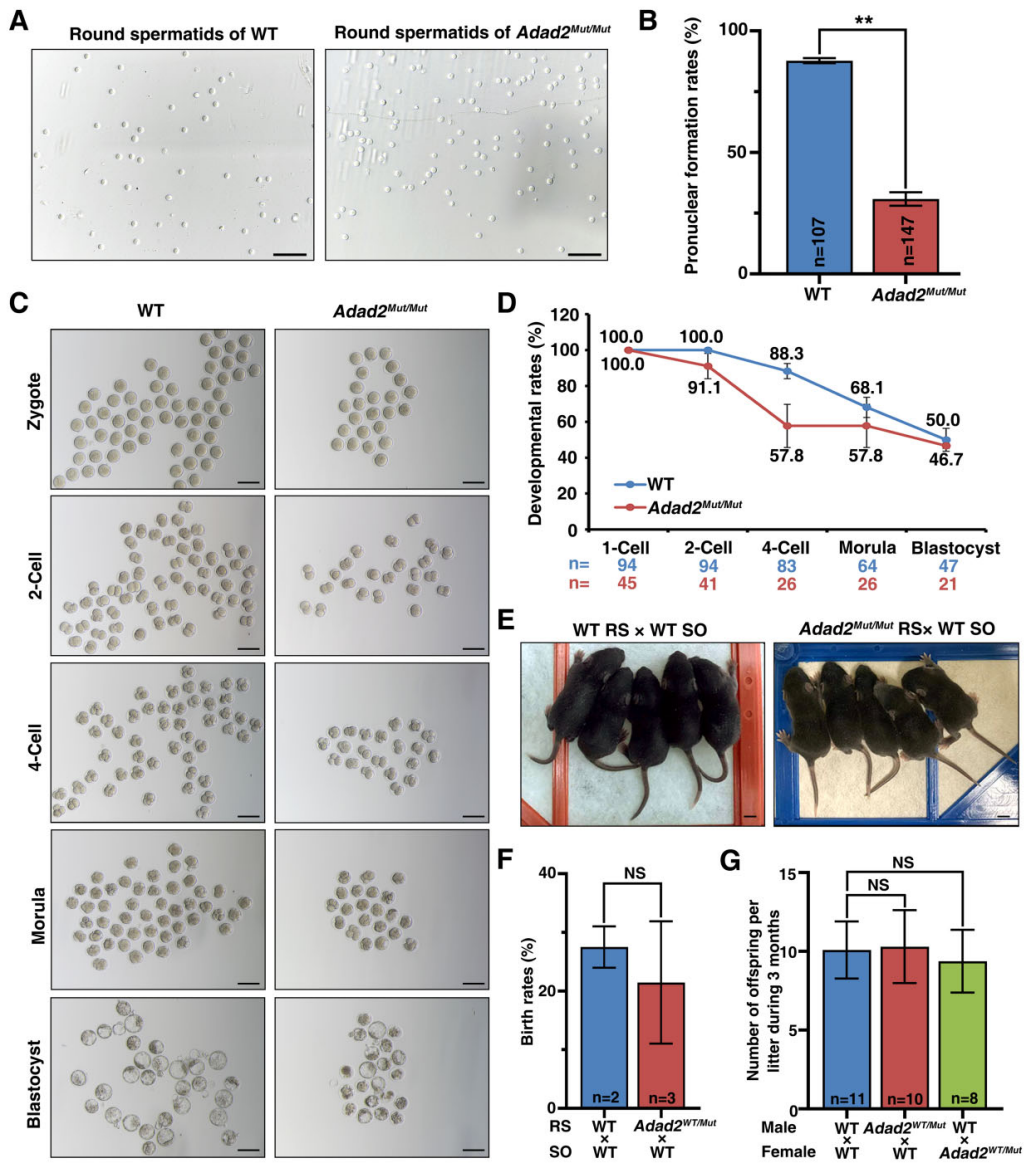
**Infertile *Adad2^Mut/Mut^* male mice could produce healthy offspring through round spermatid injection (ROSI).** (**A**) Images of round spermatids collected by fluorescence activated cell sorting. WT, wild-type. Scale bars, 50 μm. (**B**) Pronuclear formation rates after ROSI. The data are shown as mean ± SD. **P < 0.01, unpaired *t* test. (**C**) The *in vitro* preimplantation development of WT oocytes fertilized with round spermatids of WT and *Adad2^Mut/Mut^*. Scale bars, 100 μm. (**D**) Preimplantation development of ROSI-generated embryos at the indicated stages. n, number of evaluated embryos. Error bars, SD. (**E**) Representative images of healthy pups derived by ROSI. RS, round spermatids. SO, stimulated oocytes. (**F**) The birth rate of ROSI-generated pups. Birth rate equals to the number of full-term pups delivered divided by the number of two-cell stage embryos transferred. Data were presented as mean ± SD. NS, no significance (unpaired *t* test). n, rounds of ROSI repeated using WT and *Adad2^Mut/Mut^* spermatids, respectively. RS, round spermatid; SO, stimulated oocyte. (**G**) Fertility test of ROSI-derived progeny. Four WT, four *Adad2^WT/Mut^* male mice, and six *Adad2^WT/Mut^* female mice were involved in the fertility test. Each male mouse was mated with two females for 3 months. Data were presented as mean ± SD. NS, no significance (unpaired t test). n, the number of litters produced by each group.

Since the *Adad2^Mut/Mut^* mice model was observed to effectively represent the spermatogenic failure in the *ADAD2* mutation-harboring patients, we explored potential therapeutic approaches for *ADAD2*-related NOA in the *Adad2^Mut/Mut^* mice. As elongated spermatids or spermatozoa were extremely rare in the *Adad2^Mut/Mut^* mice, we attempted ROSI with round spermatids in stimulated WT oocytes (Fig. 4A). Although the round spermatids of the *Adad2^Mut/Mut^* mice exhibited a significantly lower fertilization rate (87.7 ± 1.06% in WT versus 30.85 ± 2.76% in *Adad2^Mut/Mut^*) (Fig. 4B), the *ex vivo* development from zygote to blastocyst stage was comparable between the WT and *Adad2^Mut/Mut^* spermatids (Fig. 4C and D). We next transplanted the two-cell stage embryos derived from the ROSI into pseudo-pregnant females. Inspiringly, full-term viable *Adad2^WT/Mut^* pups were delivered, and their birth rate was close to that obtained from the WT round spermatids (Fig. 4E and F). Furthermore, these heterozygous offspring (Supplementary Fig. S5) did not show any overt developmental defects and had normal fertility as adults (Fig. 4G). Taken together, our results suggested that ROSI could be a potential treatment for male infertility due to *Adad2* mutations.

## Discussion

In this study, we identified three recessive *ADAD2* mutations in six idiopathic NOA-affected men from three unrelated Pakistani families (Fig. 1A). The *ADAD2* mutations were predicted to be pathogenic from the WES data combined with strict mutation screening criteria (Supplementary Fig. S1 and Table S4). MT1/MT2 significantly reduced the testicular levels of the ADAD2 protein and produced defects in spermiogenesis similar to those seen in *Adad2*-null mice (Fig. 2). In addition, the *Adad2^Mut/Mut^* mouse model harboring MT3 had no sperm in the epididymides, suggesting a correspondence with the spermatogenic deficiency of *ADAD2*-mutant patients (Fig. 3A). The good symptomatic consistency between the patients and the mouse model suggests a causal relationship between *ADAD2* mutations and human NOA.

The ADAD2 protein is mainly expressed in spermatocytes in humans (Fig. 2B) and mice (Snyder *et al.*, 2020). The ADAD2 protein levels were observed to be dramatically lower in patients carrying MT1 or MT2 mutations (Fig. 2A), leading to defects in spermiogenesis (Fig. 2C), while spermatogonial proliferation and differentiation were not affected. The MT3 mutation (recapitulated by *Adad2^Mut/Mut^* mice) potentially results in a truncated ADAD2 protein, predicted to be 60% shorter than the full-length protein and rapidly degraded before the diplonema phase. Spermatogenic analyses of the *Adad2^Mut/Mut^* mice revealed a reduction in testicular size (Supplementary Fig. S3A and B), defective differentiation of round spermatids to elongated spermatids (Supplementary Fig. S3E), and a complete absence of spermatozoa in the epididymides (Fig. 3A; Supplementary Fig. S3C); this corresponds with the findings in *Adad2^ko^* mice (Snyder *et al.*, 2020). Therefore, the evidence of the comparable spermatogenic defects between patients with the MT1, MT2, and MT3 mutations, the *Adad2^Mut/Mut^* mice mimicking the MT3 mutation, and the reported findings on the *Adad2^ko^* mouse indicate that mutations in *ADAD2* lead to a loss-of-function effect. Taken together, we suggest that ADAD2 is dispensable for spermatogonia development in humans but has a conserved role in mammalian spermiogenesis.

*Adad2^Mut/Mut^* mice with multiple HP1a foci showed defective heterochromatin remodeling in round spermatids (Fig. 3D and E) together with significantly reduced levels of H3K4me2 marking (Fig. 3B and C). H3K4me2 is required for sperm-specific protamination (Godmann *et al.*, 2007). Reduced H3K4me2 may impede histone differentiation to protamine resulting in malformed elongated spermatids (Chukrallah *et al.*, 2022). Thus, we hypothesize that elongating/elongated spermatids in *Adad2^Mut/Mut^* mice were eliminated due to faulty chromatin remodeling and morphological abnormalities that originated in round spermatids. However, further studies are required to clearly understand the molecular mechanism of *ADAD2* mutation-induced spermatogenic failure.

In *Adad2^Mut/Mut^* mice, we observed very few elongated spermatids or spermatozoa, suggesting that the frameshift or missense variants of *ADAD2*, located in the adenosine deaminase domain, are associated with a good prognosis for testicular sperm extraction, and that the retrieved sperm might be recovered and used for ICSI. However, a recent report described a patient with asthenoteratozoospermia carrying a homozygous missense mutation of *ADAD2* (c.1381C>T, p.R461W), located in the adenosine deaminase domain, the same domain as our MT1 (MT1: c.G829T, p.G277C) and MT2 (c.G1192A, p.D398N), who failed to cause pregnancy even after ICSI treatment (Dai *et al.*, 2023), suggesting that the poor-quality spermatozoa from the *ADAD2* mutant patient were not sufficient to father a biological child. Therefore, ROSI might be an alternative therapy for patients harboring *ADAD2* mutations. Although round spermatids from *Adad2^Mut/Mut^* mice had a lower fertilization rate (Fig. 4B), comparable *ex vivo* (Fig. 4C and D) and *in vivo* (Fig. 4E and F) embryogenesis capacities were observed between WT and *Adad2^Mut/Mut^*-derived zygotes, suggesting that ADAD2 protein is involved in spermiogenesis and fertilization but not in early embryogenesis. The *Adad2^WT/Mut^* progeny derived from ROSI were viable, healthy and had normal fertility (Fig. 4G), indicating that ROSI can be a feasible treatment for infertile *Adad2^Mut/Mut^* mice. In the clinic, a ROSI-related concern is whether the inadequate replacement of histones by protamine in round spermatids would lead to epigenetic modification(s) in the paternal genome, adversely affecting the next generation (Siklenka *et al.*, 2015). Promisingly, in 2018, a tracking survey of 90 babies born from ROSI showed no significant differences in physical and cognitive development during the first two years of life compared with naturally conceived infants (Tanaka *et al.*, 2018). A recent study on the fetuses and placentae of embryonic day 11.5 mice also showed that the overall transcriptomic profiles and general methylation levels were similar between ROSI and ICSI-produced pups (Zhu *et al.*, 2021). These findings enhance our understanding of ROSI and provide valuable clues for the clinical application of ROSI.

*ADAD2* variants appear to be more frequent in Asian populations (Supplementary Fig. S6), especially the frameshift variant which has a frequency of 1 in 1420 (Supplementary Fig. S6). We propose that this could be due to a founder effect since *ADAD2* variants originated from a subset of ancestors of the Pashtun ethnic group. Notably, *ADAD2* is specifically expressed in the testes and *ADAD2* variants are deleterious in men but not in women. Hence, *ADAD2* mutations can be silent in the population in a heterozygous state in men or homozygous in women and may thus be less subject to selection pressure. The cultural practices of endogamy, polygyny and consanguineous marriage would allow the passage and expansion of this variant over generations within specific populations, leading to relatively high risks of male infertility, as seen in the Pakistani population. Apart from the *ADAD2* gene, several pathogenic variants associated with male infertility in other genes have also been reported, many of which appear to be especially prevalent in specific populations. These include the homozygous frameshift variant c.676dup in *M1AP* which was identified in multiple non-Finnish Europeans. This mutation is relatively prevalent in European populations and most likely originated from a founder mutation (Wyrwoll *et al.*, 2020). Identifying the existence of population-specific disease alleles is not only valuable for estimating the recurrence risks of related diseases in specific populations, but also provides precious resources for understanding the genetic causes of such diseases.

In summary, we identified a function of *ADAD2* in human spermiogenesis and verified a causal relationship between *ADAD2* mutations in human NOA. Inspiringly, ROSI helped produce healthy offspring from infertile *Adad2^Mut/Mut^* mice that had abnormal heterochromatin organization in round spermatids. Our work provides preliminary clues for genetic counselling of *ADAD2* mutation-associated male infertility.

## Supplementary Material

hoad022_Supplementary_Figs

hoad022_Supplementary_Tables

## Data Availability

The data used to support the findings of this study are available from the corresponding author upon request.
