# Supplementary material for: Biallelic mutations in RNA-binding protein ADAD2 cause spermiogenic failure and non-obstructive azoospermia in humans
Source: Hum Reprod Open. 2023 May 18;2023(3):hoad022. doi: 10.1093/hropen/hoad022 (PMC10266965; doi:10.1093/hropen/hoad022)
Supplement: hoad022_Supplementary_Figs [file hoad022_supplementary_figs.docx]

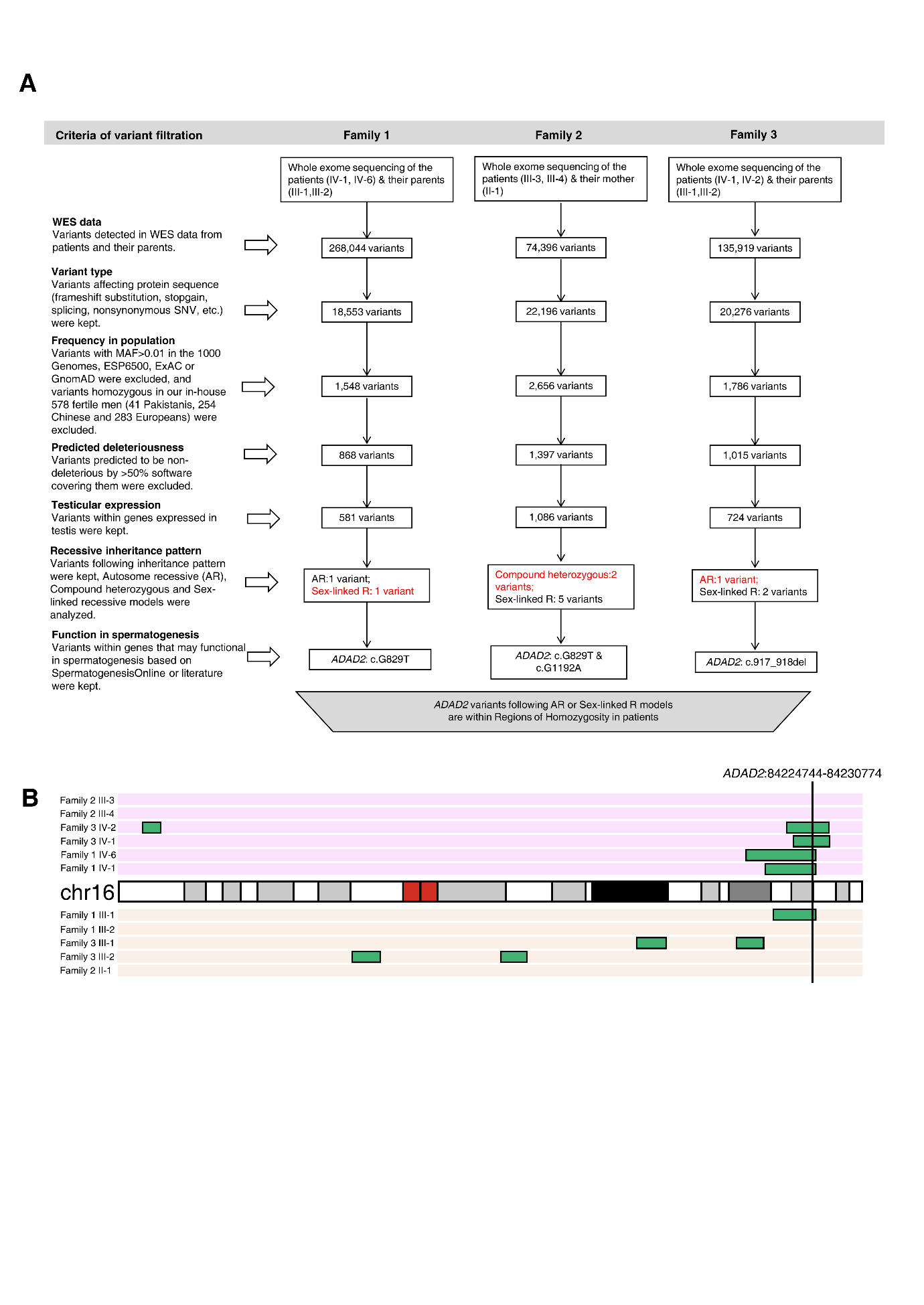


**Supplementary Figure S1 The workflow for whole-exome sequencing data analysis.**

(A) The flowchart shows the strategy to filter the candidate variants for three Pakistani families. The inheritance patterns of *ADAD2* variants are marked in red. MAF, minor allele frequency; SNV, single-nucleotide variant; R, recessive.

(B) Homozygosity mapping analysis for individuals carrying *ADAD2* mutation and their parents. The red bar indicates centromere of chromosome 16. The green bars indicate Runs of Homozygosity (RoH) regions and the black vertical line represents the site of *ADAD2* gene. The *ADAD2* gene is located in RoH regions for patients from family 1 and 3, and the mother (III-1) in family 1. While patients from family 2, father (III-2) in family 1, mother (II-1) in family2, and parents (III-1 and III-2) in family 3 does not share the RoH regions in *ADAD2* gene.


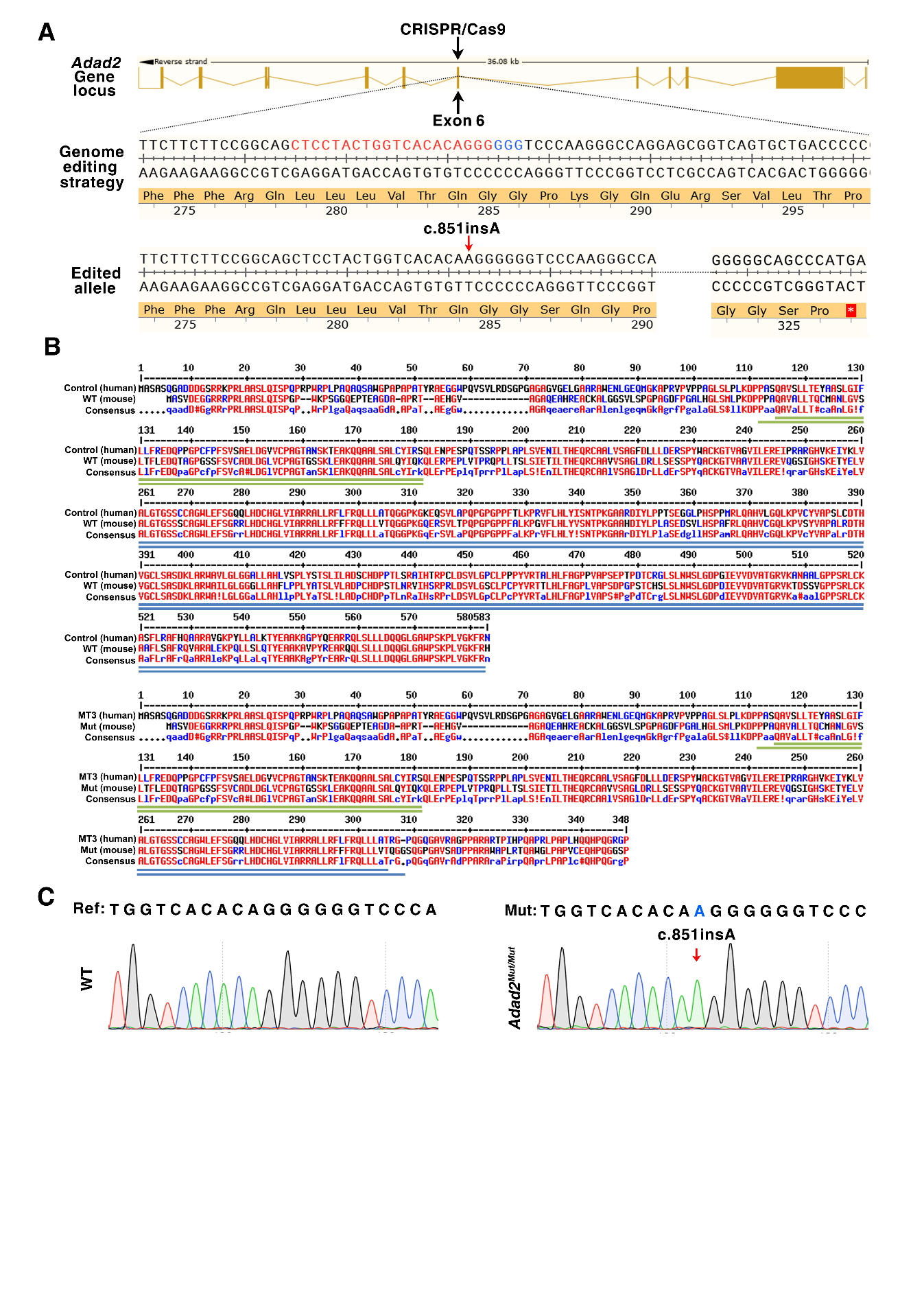


**Supplementary Figure S2 Generation *of Adad2^Mut/Mut^* mouse model.**

(A) Schematic diagram of *Adad2^Mut/Mut^* mice targeting strategy by CRISPR/Cas9. The red nucleotide sequence indicates the single guide RNA (sgRNA) targeting sequence and blue nucleotide sequence indicates protospacer adjacent motif. A base pair insertion (red arrow) introduces a premature stop codon (red asterisk).

(B) The alignment of the normal and mutated ADAD2 protein sequences in humans and mice. ADAD2 residues that are identical between humans and mice are shown in red and uppercase letters in the consensus line. Residues that are highly similar between human and mouse are indicated by red symbols (!, any one of I and V; #, any one of N, D, and E). Non-conserved residues are shown in blue or marked with asterisks in the consensus line. The green line highlights the double-stranded RNA binding (DBRM) domain of ADAD2. The blue line highlights the adenosine deaminase domain of ADAD2. The alignment was performed using the online software MultAlin (<http://multalin.toulouse.inra.fr/multalin/multalin.html>).

(C) Validation of *Adad2^Mut/Mut^* mice by genomic DNA sequencing. The inserted nucleotide is labeled in blue. The red arrow indicates the mutation site.


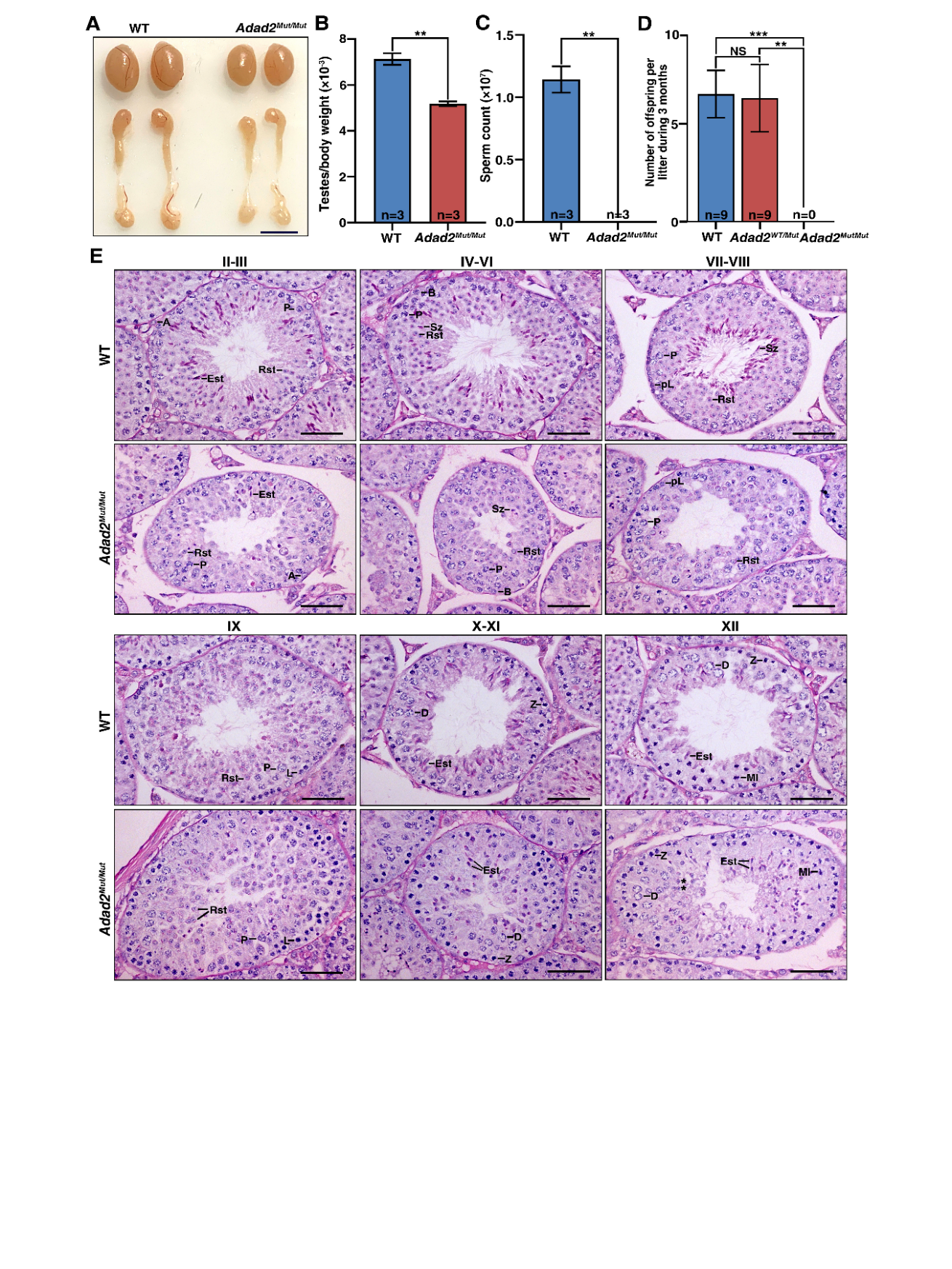


**Supplementary Figure S3 The *Adad2^Mut/Mut^* mice are infertile due to spermiogenesis failure.**

(A-B) Testicular and epididymal size (A) and testes-body weight ratio (B) of 8-week-old wild-type (WT) and *Adad2^Mut/Mut^* littermates. Scale bar, 5 mm. n, the number of mice analyzed in each group. Data were presented as mean ± standard deviation (SD). **P < 0.01, unpaired t test.

(C) Epidydimal sperm count of 8-week-old WT and *Adad2^Mut/Mut^* littermates. n, the number of mice analyzed in each group. Data were presented as mean ± SD. **P < 0.01, unpaired t test.

(D) Fertility test was performed by mating each 8-week-old male mouse with two 8-week-old females for 3 months. A total of three WT, three *Adad2^WT/Mut^* and four *Adad2^Mut/Mut^* male mice were examined. n, the number of litters produced by each group. Data were presented as mean ± SD. **P < 0.01, ***P < 0.001, NS, no significance, unpaired t test.

(E) Periodic acid-Schiff (PAS)-stained testicular sections of 8-week-old WT and *Adad2^Mut/Mut^* littermates. The identification of 12 epithelial stages were based on germ cell types, acrosome and nuclear morphology of spermatids (Ahmed and de Rooij, 2009; Meistrich and Hess, 2013). Asterisks indicate the round spermatids without elongation in *Adad2^Mut/Mut^.* A, type A spermatogonia; B, type B spermatogonia; pL, preleptonema; L, leptotene; Z,zygotene; P, pachytene; D, diplotene; MI, metaphase I; Rst, round spermatid; Est, elongating spermatid (in stage IX-XI tubules) or elongated spermatid (in stage XII and I-III tubules); Sz, spermatozoa. Scale bars, 50 μm.


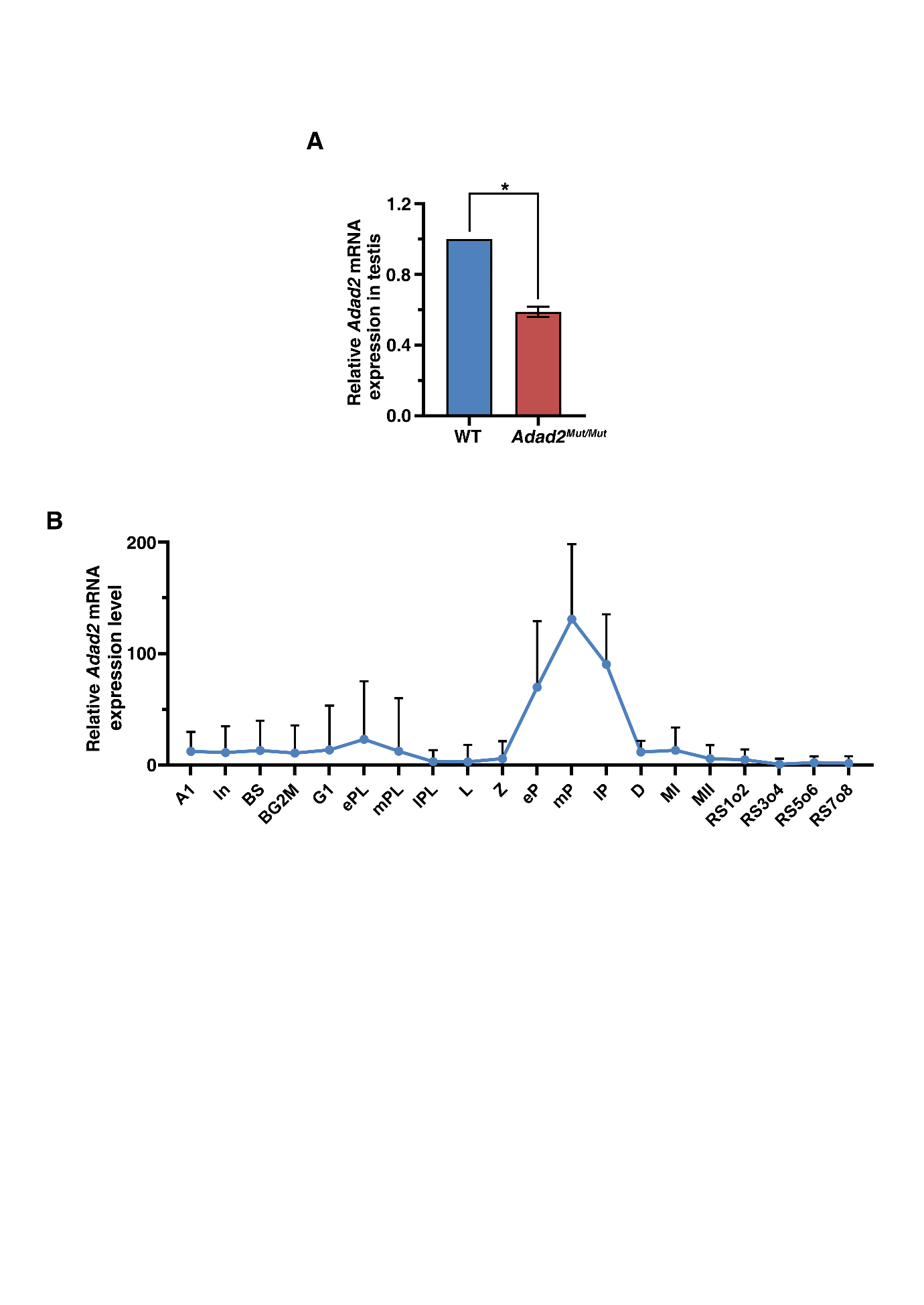


**Supplementary Figure S4 Expression pattern of ADAD2 in humans and mice.**

(A) Quantitative PCR analyses of the relative *Adad2* expression in the testes from the 3-week-old wild-type (WT) and *Adad2^Mut/Mut^* littermates. Data were obtained from three experiments and presented as mean ± standard deviation (SD). *P < 0.05, unpaired t-test. *Actb* was used as an internal control.

(B) Dynamic expression of *Adad2* mRNA during mouse spermatogenesis. The data was acquired from online single-cell RNA-seq database (<http://www.ncbi.nlm.nih.gov/gen/>, accession ID: GSE107644). A1, type A1 spermatogonia; In, intermediate spermatogonia; BS, S phase type B spermatogonia; BG2M, G2/M phase type B spermatogonia; G1, G1 phase preleptotene; ePL, early S phase preleptotene; mPL, middle S phase preleptotene; lPL, late S phase preleptotene; L, leptotene; Z, zygotene; eP, early pachytene; mP, middle pachytene; lP, late pachytene; D, diplotene; MI, metaphase I; MII, metaphase II; RS1o2, steps 1–2 spermatids; RS3o4, steps 3–4 spermatids; RS5o6, steps 5–6 spermatids; RS7o8, steps 7–8 spermatids.


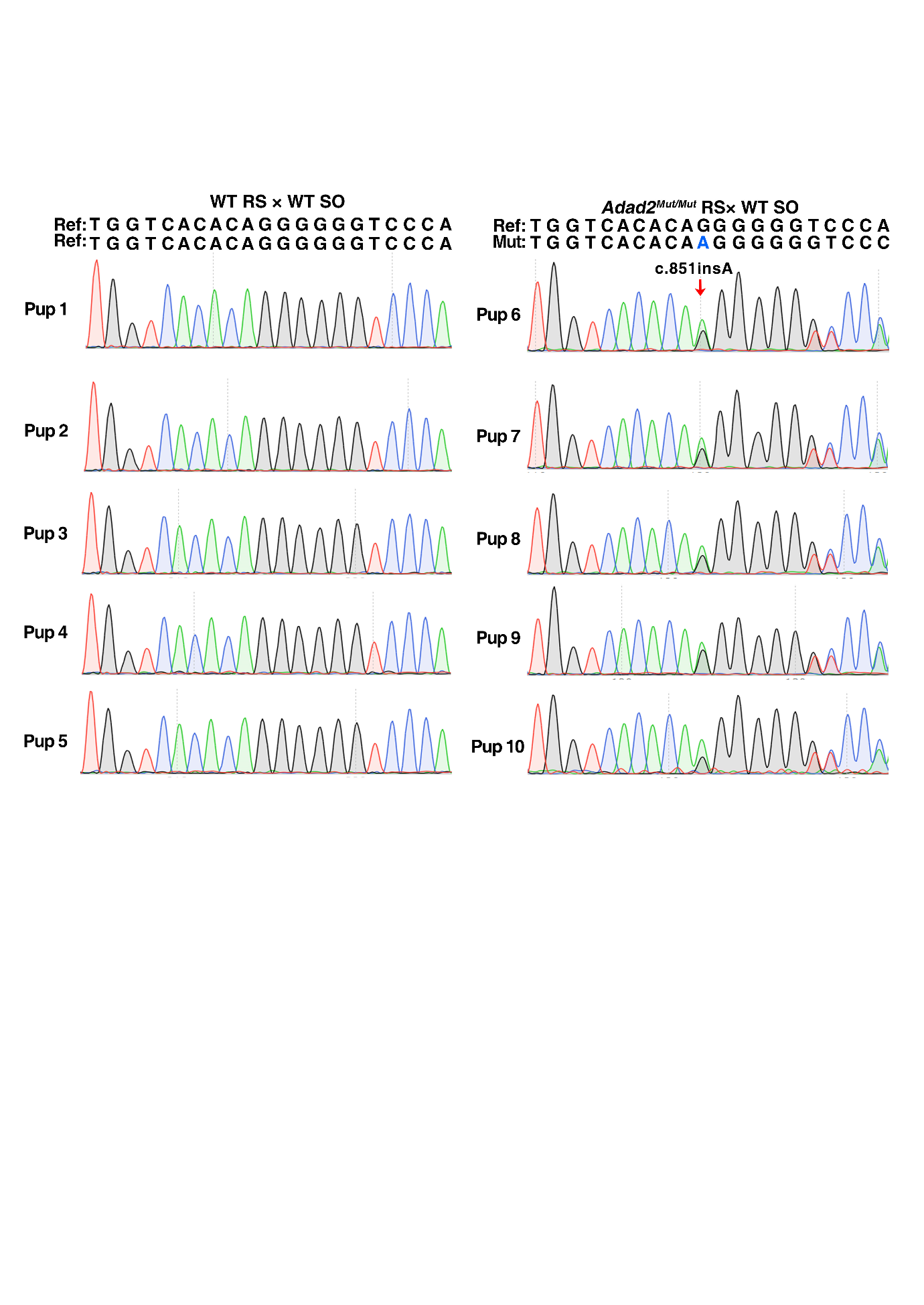


**Supplementary Figure S5 Genotypes of pups derived by round spermatid injection (ROSI).** WT, wild-type; RS, round spermatid; SO, stimulated oocyte. The inserted nucleotide is labeled in blue and the red arrow indicates the mutation site.


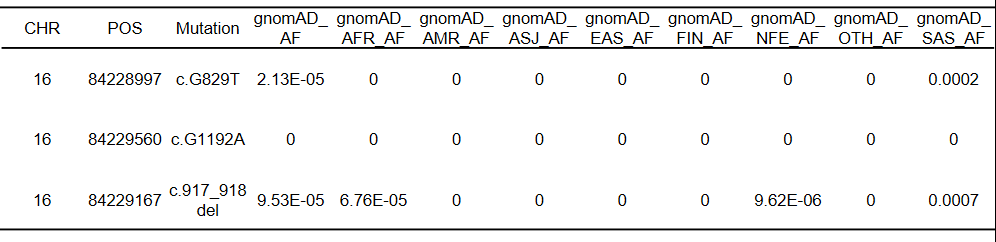


**Supplementary Figure S6 The allele frequency (AF) of *ADAD2* variants for specific populations in the Genome Aggregation Database (gnomAD).** CHR, chromosome; POS, position; AFR, African population; AMR, Admixed American population; ASJ, Ashkenazi Jewish; EAS, East Asian; FIN, Finnish; NFE, Non-Finnish European; OTH, Other (population not assigned); SAS, South Asian.

**References:**

Ahmed EA, de Rooij DG. Staging of mouse seminiferous tubule cross-sections. *Methods Mol Biol* 2009;**558**:263-277.

Meistrich ML, Hess RA. Assessment of spermatogenesis through staging of seminiferous tubules. *Methods Mol Biol* 2013;**927**:299-307.
