# Supplementary material for: Biallelic mutations in RNA-binding protein ADAD2 cause spermiogenic failure and non-obstructive azoospermia in humans
Source: Hum Reprod Open. 2023 May 18;2023(3):hoad022. doi: 10.1093/hropen/hoad022 (PMC10266965; doi:10.1093/hropen/hoad022)
Supplement: hoad022_Supplementary_Tables [file hoad022_supplementary_tables.docx]

| **Supplementary Table S1** Primers used in this study. | | | |
| --- | --- | --- | --- |
| **Information** | **Name** | **Sequence (5'- 3')** | **Product size (bp)** |
| Primers for human gDNA amplification and Sanger sequencing | hADAD2-MT1-F | GTCATCCTGGAGAGGGGTAG | Control: 366  Mutant: 366 |
|  | hADAD2-MT1-R | TGCCGGAACAAGAACCTAGG |  |
|  | hADAD2-MT2-F | TCAGAAGAGCAGCCCTGAAG | Control: 349  Mutant: 349 |
|  | hADAD2-MT2-R | AAGGGACAGGACAAGGTGAG |  |
|  | hADAD2-MT3-F | ATCTACAAGCTGGTGGCTCT | Control: 351  Mutant: 349 |
|  | hADAD2-MT3-R | CCTTGGGGGTGTTGCTGATG |  |
| Primers for genotyping  of the *Adad2* mutant mice | mAdad2-851insA-F | CCAAAGAGACCTATGAGCTG | Control: 445  Mutant: 446 |
|  | mAdad2-851insA-R | CCAGAATCAGGGTACTCCTT |  |
| Primers used for qPCR | mAdad2-qRT1-CF1 | AACCTGGGTGTCTCTCTCAC | 173 |
|  | mAdad2-qRT-CR1 | AGCTGCTTCTGGATGTACTG |  |
|  | mActb-qRT-CF | TGGAGAAGATCTGGCACCAC | 167 |
|  | mActb-qRT-CR | CTGGATGGCTACGTACATGG |  |

| **Supplementary Table S2** Image acquisition parameters in this study. | | | | | | |  |  |  |  |  |  |
| --- | --- | --- | --- | --- | --- | --- | --- | --- | --- | --- | --- | --- |
|  | **Microscope type** | **Objective lens magnification** | **Pixel size** | **NA** | **Excitation/Emission wavelength (ADAD2)** | **Exposure time (ADAD2)** | **Excitation/Emission wavelength (SYCP3)** | **Exposure time (SYCP3)** | **Excitation/Emission wavelength (Hoechst)** | **Exposure time (Hoechst)** |  |  |
| **Figure 2B-control** | Olympus BX53 Microscope | 40X | 2048x2048 | 1.4 | 578/603 nm | 188.515ms | 498/519 nm | 251.215ms | 350/460 nm | 10.815ms |  |  |
| **Figure 2B-Family 1 IV-1** | Olympus BX53 Microscope | 40X | 2048x2048 | 1.4 | 578/603 nm | 188.515ms | 498/519 nm | 99.215ms | 350/460 nm | 4.815 ms |  |  |
| **Figure 2B-Family 2 III-3** | Olympus BX53 Microscope | 40X | 2048x2048 | 1.4 | 578/603 nm | 188.515ms | 498/519 nm | 230 ms | 350/460 nm | 19.52ms |  |  |
|  |  |  |  |  |  |  |  |  |  |  |  |  |
|  | **Microscope type** | **Objective lens magnification** | **Pixel size** | **NA** | **Exposure time** |  |  |  |  |  |  |  |
| **Figure 2C-control** | Nikon ECLIPSE 80i Microscope | 40X | 1280x1024 | 1.4 | 15 ms |  |  |  |  |  |  |  |
| **Figure 2C-Family 1 IV-1** | Nikon ECLIPSE 80i Microscope | 40X | 1280x1024 | 1.4 | 15 ms |  |  |  |  |  |  |  |
| **Figure 2C-Family 2 III-3** | Nikon ECLIPSE 80i Microscope | 40X | 1280x1024 | 1.4 | 17 ms |  |  |  |  |  |  |  |
|  |  |  |  |  |  |  |  |  |  |  |  |  |
|  | **Microscope type** | **Objective lens magnification** | **Pixel size** | **NA** | **Exposure time** |  |  |  |  |  |  |  |
| **Figure 3A-WT-VII-VIII** | Nikon ECLIPSE 80i Microscope | 40X | 1280x1024 | 1.4 | 15 ms |  |  |  |  |  |  |  |
| **Figure 3A*-Adad2*^Mut/Mut^-VII-VIII** | Nikon ECLIPSE 80i Microscope | 40X | 1280x1024 | 1.4 | 18 ms |  |  |  |  |  |  |  |
| **Figure 3A-WT-epididymis** | Nikon ECLIPSE 80i Microscope | 20X | 1280x1024 | 1.4 | 6 ms |  |  |  |  |  |  |  |
| **Figure 3A-*Adad2*^Mut/Mut^-epididymis** | Nikon ECLIPSE 80i Microscope | 20X | 1280x1024 | 1.4 | 6 ms |  |  |  |  |  |  |  |
|  |  |  |  |  |  |  |  |  |  |  |  |  |
|  | **Microscope type** | **Objective lens magnification** | **Pixel size** | **NA** | **Excitation/Emission wavelength (HP1α)** | **Exposure time (HP1α)** | **Excitation/Emission wavelength (SYCP3)** | **Exposure time (SYCP3)** | **Excitation/Emission wavelength (Hoechst)** | **Exposure time (Hoechst)** |  |  |
| **Figure 3D-WT** | Olympus BX53 Microscope | 40X | 2048x2048 | 1.4 | 554/568 nm | 275.215 ms | 498/519 nm | 718.815 ms | 350/460 nm | 12.415 ms |  |  |
| **Figure 3D-*Adad2*^Mut/Mut^** | Olympus BX53 Microscope | 40X | 2048x2048 | 1.4 | 554/568 nm | 275.215 ms | 498/519 nm | 548.415 ms | 350/460 nm | 32.815 ms |  |  |
|  |  |  |  |  |  |  |  |  |  |  |  |  |
|  | **Microscope type** | **Objective lens magnification** | **Pixel size** | **NA** | **Excitation/Emission wavelength (ADAD2)** | **Exposure time (ADAD2)** | **Excitation/Emission wavelength (PNA)** | **Exposure time (PNA)** | **Excitation/Emission wavelength (γH2AX)** | **Exposure time (γH2AX)** | **Excitation/Emission wavelength (Hoechst)** | **Exposure time (Hoechst)** |
| **Figure 3F-I** | Olympus BX53 Microscope | 40X | 2048x2048 | 1.4 | 578/603 nm | 200.015 ms | 498/519 nm | 12.615 ms | 650/671 nm | 200.015 ms | 350/460 nm | 12.615 ms |
| **Figure 3F-II-III** | Olympus BX53 Microscope | 40X | 2048x2048 | 1.4 | 578/603 nm | 200.015 ms | 498/519 nm | 44.915 ms | 650/671 nm | 300.015 ms | 350/460 nm | 17.815 ms |
| **Figure 3F-IV** | Olympus BX53 Microscope | 40X | 2048x2048 | 1.4 | 578/603 nm | 200.015 ms | 498/519 nm | 46.515 ms | 650/671 nm | 400.015 ms | 350/460 nm | 24.515 ms |
| **Figure 3F-V-VI** | Olympus BX53 Microscope | 40X | 2048x2048 | 1.4 | 578/603 nm | 200.015 ms | 498/519 nm | 28.315 ms | 650/671 nm | 200.015 ms | 350/460 nm | 12.915 ms |
| **Figure 3F-VII-VIII** | Olympus BX53 Microscope | 40X | 2048x2048 | 1.4 | 578/603 nm | 200.015 ms | 498/519 nm | 41.815 ms | 650/671 nm | 400.015 ms | 350/460 nm | 19.815 ms |
| **Figure 3F-IX** | Olympus BX53 Microscope | 40X | 2048x2048 | 1.4 | 578/603 nm | 200.015 ms | 498/519 nm | 51.515 ms | 650/671 nm | 400.015 ms | 350/460 nm | 30.015 ms |
| **Figure 3F-X-XI** | Olympus BX53 Microscope | 40X | 2048x2048 | 1.4 | 578/603 nm | 200.015 ms | 498/519 nm | 88.515 ms | 650/671 nm | 350.015 ms | 350/460 nm | 24.515 ms |
| **Figure 3F-XII** | Olympus BX53 Microscope | 40X | 2048x2048 | 1.4 | 578/603 nm | 200.015 ms | 498/519 nm | 46.515 ms | 650/671 nm | 400.015 ms | 350/460 nm | 24.215 ms |
|  |  |  |  |  |  |  |  |  |  |  |  |  |
|  | **Microscope type** | **Objective lens magnification** | **Pixel size** | **NA** | **Excitation/Emission wavelength (ADAD2)** | **Exposure time (ADAD2)** | **Excitation/Emission wavelength (PNA)** | **Exposure time (PNA)** | **Excitation/Emission wavelength (γH2AX)** | **Exposure time (γH2AX)** | **Excitation/Emission wavelength (Hoechst)** | **Exposure time (Hoechst)** |
| **Figure 3G-IV-VI** | Olympus BX53 Microscope | 40X | 2048x2048 | 1.4 | 578/603 nm | 200.015 ms | 498/519 nm | 24.815 ms | 650/671 nm | 100.015 ms | 350/460 nm | 26.815 ms |
| **Figure 3G-IX-XI** | Olympus BX53 Microscope | 40X | 2048x2048 | 1.4 | 578/603 nm | 200.015 ms | 498/519 nm | 89.215 ms | 650/671 nm | 400.015 ms | 350/460 nm | 30.015 ms |
| **Figure 3G-XII** | Olympus BX53 Microscope | 40X | 2048x2048 | 1.4 | 578/603 nm | 200.015 ms | 498/519 nm | 123.015 ms | 650/671 nm | 200.015 ms | 350/460 nm | 26.015 ms |
|  |  |  |  |  |  |  |  |  |  |  |  |  |
|  | **Microscope type** | **Objective lens magnification** | **Pixel size** | **NA** | **Exposure time** |  |  |  |  |  |  |  |
| **Figure 4A-WT** | Nikon Ti2-U Microscope | 10X | 1636x1088 | 1.4 | 224 ms |  |  |  |  |  |  |  |
| **Figure 4A-*Adad2*^Mut/Mut^** | Nikon Ti2-U Microscope | 10X | 1636x1088 | 1.4 | 1000 ms |  |  |  |  |  |  |  |
|  |  |  |  |  |  |  |  |  |  |  |  |  |
|  | **Microscope type** | **Objective lens magnification** | **Pixel size** | **NA** | **Exposure time** |  |  |  |  |  |  |  |
| **Figure 4C-WT-Zygote** | Nikon Ti2-U Microscope | 10X | 1636x1088 | 1.4 | 389 ms |  |  |  |  |  |  |  |
| **Figure 4C-*Adad2*^Mut/Mut^-Zygote** | Nikon Ti2-U Microscope | 10X | 1636x1088 | 1.4 | 400 ms |  |  |  |  |  |  |  |
| **Figure 4C-WT-2-Cell** | Nikon Ti2-U Microscope | 10X | 1636x1088 | 1.4 | 193 ms |  |  |  |  |  |  |  |
| **Figure 4C-*Adad2*^Mut/Mut^-2-Cell** | Nikon Ti2-U Microscope | 10X | 1636x1088 | 1.4 | 202 ms |  |  |  |  |  |  |  |
| **Figure 4C-WT-4-Cell** | Nikon Ti2-U Microscope | 10X | 1636x1088 | 1.4 | 375 ms |  |  |  |  |  |  |  |
| **Figure 4C-*Adad2*^Mut/Mut^-4-Cell** | Nikon Ti2-U Microscope | 10X | 1636x1088 | 1.4 | 401 ms |  |  |  |  |  |  |  |
| **Figure 4C-WT-Morula** | Nikon Ti2-U Microscope | 10X | 1636x1088 | 1.4 | 231 ms |  |  |  |  |  |  |  |
| **Figure 4C-*Adad2*^Mut/Mut^-Morula** | Nikon Ti2-U Microscope | 10X | 1636x1088 | 1.4 | 246 ms |  |  |  |  |  |  |  |
| **Figure 4C-WT-Blastocyst** | Nikon Ti2-U Microscope | 10X | 1636x1088 | 1.4 | 188 ms |  |  |  |  |  |  |  |
| **Figure 4C-*Adad2*^Mut/Mut^-Blastocyst** | Nikon Ti2-U Microscope | 10X | 1636x1088 | 1.4 | 199 ms |  |  |  |  |  |  |  |
|  |  |  |  |  |  |  |  |  |  |  |  |  |
|  | **Microscope type** | **Objective lens magnification** | **Pixel size** | **NA** | **Exposure time** |  |  |  |  |  |  |  |
| **Supplementary Figure S3E-WT-II-III** | Nikon ECLIPSE 80i Microscope | 40X | 1280x1024 | 1.4 | 15 ms |  |  |  |  |  |  |  |
| **Supplementary Figure S3E-*Adad2*^Mut/Mut^-II-III** | Nikon ECLIPSE 80i Microscope | 40X | 1280x1024 | 1.4 | 8 ms |  |  |  |  |  |  |  |
| **Supplementary Figure S3E-WT-IV-VI** | Nikon ECLIPSE 80i Microscope | 40X | 1280x1024 | 1.4 | 15 ms |  |  |  |  |  |  |  |
| **Supplementary Figure S3E-*Adad2*^Mut/Mut^-IV-VI** | Nikon ECLIPSE 80i Microscope | 40X | 1280x1024 | 1.4 | 7 ms |  |  |  |  |  |  |  |
| **Supplementary Figure S3E-WT-VII-VIII** | Nikon ECLIPSE 80i Microscope | 40X | 1280x1024 | 1.4 | 15 ms |  |  |  |  |  |  |  |
| **Supplementary Figure S3E-*Adad2*^Mut/Mut^-VII-VIII** | Nikon ECLIPSE 80i Microscope | 40X | 1280x1024 | 1.4 | 5 ms |  |  |  |  |  |  |  |
| **Supplementary Figure S3E-WT-IX** | Nikon ECLIPSE 80i Microscope | 40X | 1280x1024 | 1.4 | 15 ms |  |  |  |  |  |  |  |
| **Supplementary Figure S3E-*Adad2*^Mut/Mut^-IX** | Nikon ECLIPSE 80i Microscope | 40X | 1280x1024 | 1.4 | 8 ms |  |  |  |  |  |  |  |
| **Supplementary Figure S3E-WT-X-XI** | Nikon ECLIPSE 80i Microscope | 40X | 1280x1024 | 1.4 | 15 ms |  |  |  |  |  |  |  |
| **Supplementary Figure S3E-*Adad2*^Mut/Mut^-X-XI** | Nikon ECLIPSE 80i Microscope | 40X | 1280x1024 | 1.4 | 7 ms |  |  |  |  |  |  |  |
| **Supplementary Figure S3E-WT-XII** | Nikon ECLIPSE 80i Microscope | 40X | 1280x1024 | 1.4 | 16 ms |  |  |  |  |  |  |  |
| **Supplementary Figure S3E-*Adad2*^Mut/Mut^-XII** | Nikon ECLIPSE 80i Microscope | 40X | 1280x1024 | 1.4 | 8 ms |  |  |  |  |  |  |  |
|  |  |  |  |  |  |  |  |  |  |  |  |  |

| **Supplementary Table S3** Antibodies used in this study. | | | | |
| --- | --- | --- | --- | --- |
| **Primary antibodies** |  |  |  |  |
| **Antibody** | **Host** | **Producer** | **Catalog number** | **Dilution** |
| ADAD2 (for human) | Rat | Custom-made | - | IF: 1:50 |
| ADAD2 (for human) | Rabbit | Custom-made | - | WB: 1:500 |
| ADAD2 (for mouse) | Rat | Custom-made | - | IF: 1:50 |
| H3K4me2 | Rabbit | Abcam | ab7766 | WB: 1:500 |
| H3K9me3 | Rabbit | Abclonal | A2360 | WB: 1:500 |
| Histone H3 | Rabbit | Cell Signaling | 4620S | WB: 1:1000 |
| HP1α | Rabbit | Abcam | ab109028 | IF: 1:100 |
| Lectin PNA conjugated Alexa-488 | Peanut | ThermoFisher | L21409 | IF: 1:100 |
| SYCP3 (for human) | Mouse | Proteintech | 66409-1-Ig | IF: 1:100 |
| SYCP3 (or mouse) | Mouse | Abcam | ab97672 | IF: 1:100 |
| ACTB | Rabbit | Abcam | ab8227 | WB: 1:3000 |
| γH2AX | Rabbit | Novus | NB100-384 | IF:1:5000 |
| **Secondary antibodies** |  |  |  |  |
| **Antibody** | **Host** | **Company** | **Catalog number** | **Dilution** |
| Mouse (Alexa-488) | Goat | Molecular Probes | A21121 | IF: 1:100 |
| Rabbit (Alexa-555) | Donkey | Molecular Probes | A31572 | IF: 1:200 |
| Rabbit (Alexa-647) | Goat | ThermoFisher | A21244 | IF: 1:100 |
| Rat (Alexa-568) | Goat | ThermoFisher | A11077 | IF: 1:200 |
| Rabbit (HRP) | Donkey | Biolegend | 406401 | WB: 1:8000 |

| **Supplementary Table S4** Variants identified by WES following bioinformatic analysis for the Pakistani families. | | | | | | | | | | | | |
| --- | --- | --- | --- | --- | --- | --- | --- | --- | --- | --- | --- | --- |
| **Family 1** | **Recessive inheritance pattern** | **Human gene symbol** | **Mouse gene symbol** | **cDNA alteration** | **Amino acid alteration** | **Mutation type** | **Genotype** | **Ratio of software predicting deleterious** | **Number of software predicting deleterious** | **Number of software covering the variant** | **Remark** | **Reference** |
|  | Sex-linked R | *ADAD2* | *Adad2* | G829T | G277C | nonsynonymous SNV | Homozygous | 1 | 7 | 7 | 1. Mice homozygous for a knock-out allele exhibit male sterility and germ cells unable to progress beyond round spermatid. 2. mRNA exhibits testis-specific expression. | doi:10.1038/s41598-020-67834-5 |
|  | AR | *DOCK8* | *Dock8* | G3079A | V1027I | nonsynonymous SNV | Homozygous | 0.57 | 4 | 7 | 1. Mice homozygous for inactivating mutations of this gene exhibit loss of marginal zone B cells, decrease in peritoneal B1 cells and peripheral naive T cells, failure of sustained antibody response after immunization, failure of germinal center persistence, and failure of B cell affinity maturation. 2. mRNA exhibits ubiquitously expressed in various tissues. 3. Diseases associated include hyper-IgE recurrent infection syndrome. | MGI:1921396 MIM:611432 |
| **Family 2** | Compound heterozygote | *ADAD2* | *Adad2* | G829T | G277C | nonsynonymous SNV | Compound heterozygous | 1 | 7 | 7 | 1. Mice homozygous for a knock-out allele exhibit male sterility and germ cells unable to progress beyond round spermatid. 2. mRNA exhibits testis-specific expression. | doi:10.1038/s41598-020-67834-5 |
|  |  |  |  | G1192A | D398N | nonsynonymous SNV |  |  |  |  |  |  |
|  | Sex-linked R | *ATRX* | *Atrx* | 4330_4345A | N/A | nonframeshift substitution | Homozygous | 0 | 0 | 0 | 1. Mice homozygous for a floxed allele activated in different tissues at different time points can serve as a model of alpha-thalassemia/mental retardation syndrome, nondeletion type, and X-linked. 2. mRNA exhibits ubiquitously expressed in various tissues. 3. Diseases associated include alpha-thalassemia myelodysplasia syndrome, alpha-thalassemia/mental retardation syndrome, Intellectual disability-hypotonic facies syndrome. | doi:10.1101/cshperspect.a026567  MGI:103067 MIM:300032 |
|  |  | *RPL10* | *Rpl10* | T581A | L194H | nonsynonymous SNV | Homozygous | 0 | 0 | 0 | 1. X-linked intellectual disability, cerebellar hypoplasia, skeletal abnormalities, mental retardation, seizures, microcephaly, epilepsy and autism.  2. mRNA exhibits ubiquitously expressed in various tissues. | doi:10.3390/cells9112503 MGI:105943 MIM:312173 |
|  |  | *AIFM1* | *Aifm1* | G602A | R201K | nonsynonymous SNV | Homozygous | 1 | 7 | 7 | 1. Hemizygous males and homozygous females exhibit variable levels of hair loss and late-onset, progressive, neural degeneration with ataxia, tremors, and loss of cerebellar and retinal cells. The degree of hair loss and ataxia in heterozygous females correlates with the extent of X-inactivation. 2. mRNA exhibits ubiquitously expressed in various tissues. 3. Diseases associated include combined oxidative phosphorylation deficiency, cowchock syndrome, deafness, spondyloepimetaphyseal dysplasia with hypomyelinating leukodystrophy. | MGI:1349419 MIM:300169 |
|  |  | *FDFT1* | *Fdft1* | 192_198G | N/A | nonframeshift substitution | Homozygous | 0 | 0 | 0 | 1. Mice homozygous for a null mutation die around E9.5-10.5; Conditional homozygous null in which the gene is deleted specifically in oligodendrocyte and Schwann cell display dysmyelination of spinal cord and brain white matter, and showed ataxia and tremor. 2. mRNA exhibits ubiquitously expressed in various tissues. 3. Diseases associated include squalene synthase deficiency. | MGI:102706 MIM:184420 |
|  |  | *TMEM8A* | *Tmem8* |  | N/A | splicing | Homozygous | 0 | 0 | 0 | 1. Mice homozygous for a knock-out allele exhibit defects in rostral-caudal axis patterning, embryonic growth arrest, and embryonic lethality. 2. mRNA exhibits ubiquitously expressed in various tissues. | MGI:1926283 |
| **Family 3** | AR | *ADAD2* | *Adad2* | 917_918del | N/A | frameshift substitution | Homozygous | 0 | 0 | 0 | 1. Mice homozygous for a knock-out allele exhibit male sterility and germ cells unable to progress beyond round spermatid. 2. mRNA exhibits testis-specific expression. | doi:10.1038/s41598-020-67834-5 |
|  | Sex-linked R | *H2BFM* | *H2bfm* | G365T | R122L | nonsynonymous SNV | Homozygous | 0.86 | 6 | 7 | Chromosome X, core component of nucleosome. | MGI:1916639 UniProtKB:P0C1H6 |
|  |  | *LNP1* | *Lnp1* | 194delinsAT  CCTAGAAG  GCATTCTC  ATGAGGAC  CAGGAATT  CCGATGCC  GATCGTCT  GACCGTCT | N/A | nonframeshift substitution | Homozygous | 0 | 0 | 0 | Diseases associated include leukemia, acute lymphoblastic leukemia, Neurodevelopmental disorder with epilepsy and hypoplasia of the corpus callosum. | doi:10.1016/j.leukres.2015.04.014 doi:10.1158/2159-8290.CD-21-0674. MIM:610236 |
| The software used in this study was listed, Sorting Intolerant From Tolerant (SIFT), PolyPhen2 HDIV, MutationTaster, MutationAssessor, fathmm_MKL, GERP++, and SiPhy. N/A, Not available. | | | | | | | | | | | | |
